# The effects of being habitually barefoot on foot mechanics and motor performance in children and adolescents aged 6–18 years: study protocol for a multicenter cross-sectional study (Barefoot LIFE project)

**DOI:** 10.1186/s13047-016-0166-1

**Published:** 2016-09-02

**Authors:** Karsten Hollander, Babette C. van der Zwaard, Johanna Elsabe de Villiers, Klaus-Michael Braumann, Ranel Venter, Astrid Zech

**Affiliations:** 1 Department of Sports and Exercise Medicine, Institute of Human Movement Science, University of Hamburg, Hamburg, Germany; 2 Department of Sport Science, Stellenbosch University, Stellenbosch, South Africa; 3 Department of Sport Science, Friedrich Schiller University Jena, Jena, Germany

**Keywords:** Habitual barefoot, Biomechanics, Footwear, Foot arch, Foot morphology, Motor performance

## Abstract

**Background:**

Barefoot locomotion has evoked an increasing scientific interest with a controversial debate about benefits and limitations of barefoot and simulated barefoot walking and running. While most current knowledge comes from cross sectional laboratory studies, the evolutionary perspective suggests the importance of investigating the long-term effects. Observing habitually barefoot populations could fill the current gap of missing high quality longitudinal studies. Therefore, the study described in this design paper aims to investigate the effects of being habitually barefoot on foot mechanics and motor performance of children and adolescents.

**Methods:**

This study has a cross-sectional, binational design and is part of the “Barefoot Locomotion for Individual Foot- and health Enhancement (Barefoot LIFE)” project. Two large cohorts (n_(total)_ = 520) of healthy children and adolescents between 6 and 18 years of age will be included respectively in Germany and South Africa. A barefoot questionnaire will be used to determine habitually barefoot individuals. The testing will be school-based and include foot mechanical (static arch height index, dynamic arch index, foot pliability) and motor performance (coordination, speed, leg power) outcomes. Gender, BMI and level of physical activity will be considered for confounding.

**Discussion:**

The strength of this study is the comparison of two large cohorts with different footwear habits to determine long-term effects of being habitually barefoot on foot mechanics and motor performance.

**Electronic supplementary material:**

The online version of this article (doi:10.1186/s13047-016-0166-1) contains supplementary material, which is available to authorized users.

## Background

The general interest in barefoot locomotion has increased and attracted a scientific focus for more than a decade. There is an ongoing debate on the advantages and disadvantages [1, 2] with most of the knowledge coming from cross-sectional laboratory or field studies [3–5].

Numerous studies show that acute barefoot walking or running change foot strike pattern from a rearfoot strike to a mid-or forefoot strike with subsequently more ankle plantar flexion at foot strike [6–8], decreased stride length and increased stride frequency [4, 6, 7], reduced ground reaction forces [3, 6, 7] and increased range of motion (ROM) in the midfoot and MTP joints [6]. While this emphasizes the evidence for short-term effects [3, 4], the influence of long-term (habitual) barefoot locomotion on biomechanics, motor performance and injuries remains unclear [Hollander K, Heidt C, Van der Zwaard B, Braumann KM, Zech A. Long-term effects of habitual barefoot running and walking: a systematic review. Manuscript submitted for publication]. Few prospective studies which evaluated the effects of regular barefoot running interventions in habitually shod people reported no or controversial findings regarding relative injury rates [9], biomechanics [10] and running economy [11, 12].

Additionally, several cross-sectional studies have evaluated the effect of living habitually barefoot on foot posture and foot mechanics. A general consensus seems to be that habitually barefoot individuals have stronger feet and less foot and toe deformities [13–15]. The most evident difference to habitually shod individuals is that the foot of habitually barefoot individuals is wider in the forefoot region [16–18]. Furthermore, it is found that habitually barefoot feet have a higher arch [14, 15, 19], are more pliable [20] and have a reduced hallux angle [21]. However, there are several limitations to these studies that have to be mentioned. While some studies investigate habitually barefoot children [14, 15], other authors describe habitually barefoot adult populations [16, 17]. It is known that the foot and foot arch characteristics still develop during childhood and plateau in adolescence [22]. Furthermore, some of these studies are of low methodological quality [3]. Also, the differences between habitually barefoot and shod participants are not always clearly defined. And lastly, several studies use participants from different continents, ethnic backgrounds and possibly also socioeconomic backgrounds [16, 20], which influences the interpretation of the results. It has been shown that there are significant differences in foot morphology between different ethnicities and therefore ethnicity should be taken into account when assessing skeletal differences [23].

Although previous studies suggest differences in foot posture and foot mechanics between habitually barefoot and shod people, the clinical and practical relevance remain speculative. However, cross-sectional studies on short-term barefoot effects emphasize the hypothesis that regular locomotion with and without shoes influences motor performance of adults [5] and children [Hollander K, Heidt C, Van der Zwaard B, Braumann KM, Zech A. Long-term effects of habitual barefoot running and walking: a systematic review. Manuscript submitted for publication]. In this context, one may speculate whether the magnitude of such effects may increase with age (and barefoot experience).

The aim of our study is to evaluate the effect of being habitually barefoot on foot mechanics and motor performance of children and adolescents between the ages of 6–18 years. Additionally, we will evaluate if differences between habitually barefoot and shod children are age-dependent. Based on current knowledge, we hypothesize that children growing up wearing shoes regularly, have lower arches compared to their barefoot counterparts. It is also anticipated that the habitually barefoot children perform better on the barefoot motor performance tasks than the habitually shod children.

## Methods/Design

This protocol is reported according to the SPIRIT statement for improved reporting of study protocols [24].

### Study design

This is a binational multicenter, cross-sectional observational study looking at differences of foot mechanics and motor performance between habitually barefoot and habitually shod children and adolescents aged 6–18 years. Ethical approval has been obtained from the ethics committee of the medical association Hamburg (protocol number PV4971) and Stellenbosch University ethics committee (protocol number HS1153/2014). The regional separation of the recruitment is due to the obligation of footwear use in most German educational institution while in South Africa the habit of being barefoot prevails.

### Participants

After pilot testing for reliability and validity of the measurement apparatus, recruitment of participants exclusively will take place in rural and urban primary and secondary schools with no restriction to school type. In South Africa, primary school attendees are aged 6–13/14 and secondary school children are between 13/14–18 years old, their German counterparts are 6–9/10 and 9/10–18. With approval from the German and South African supervisory school authorities, schools will be randomly selected per stratum (representing a combination of district and type of school) and contacted by the principal investigators. Schools (in blocks of five primary schools and five secondary schools) will be initially contacted via email and when interested visited by the study staff for further organisation. If the school wants to participate, consent forms for all pupils (and their parents) will be provided in the appropriate language (English, Afrikaans, Xhosa or German, Additional file 1). No limit will be set per school for maximum number of participating children per age. We will strive for an equal distribution of portion of participants per school to ensure equal representation. For participation, pupils will be requested to bring along the signed consent form on the testing day.

Inclusion criteria will consist of healthy children that are physically active for at least 120 accumulative minutes per week (parent reported). Children and young adolescents between the age of 6 and 18 will be recruited for this study. Exclusion criteria will be evaluated by parent proxy report and consist of current injuries of the lower extremity, abnormal gait or any neurological or neuromuscular abnormalities likely to influence the gait.

### Testing procedure

Methodological planning stipulates all anthropometrical, foot and motor performance measurements to be performed during a physical education lesson. Prior to the testing, a physical activity questionnaire for children/adolescent (PAQ-C and PAQ-A [25, 26]) and a barefoot questionnaire will be distributed by the teachers and collected by the study staff on the testing day and briefly checked for completeness. All children bringing the signed consent form and voluntarily want to participate will be gathered and all relevant information for the testing will be given by the principal investigator. After a short warm up period (jogging for < 5 min), participants will be randomly allocated to the seven testing stations:

1. Anthropemetry
2. Foot caliper
3. Pressure plate
4. 20 m sprinting
5. Lateral jumping
6. Standing long jump
7. Backwards balancing

**Fig. 1:**
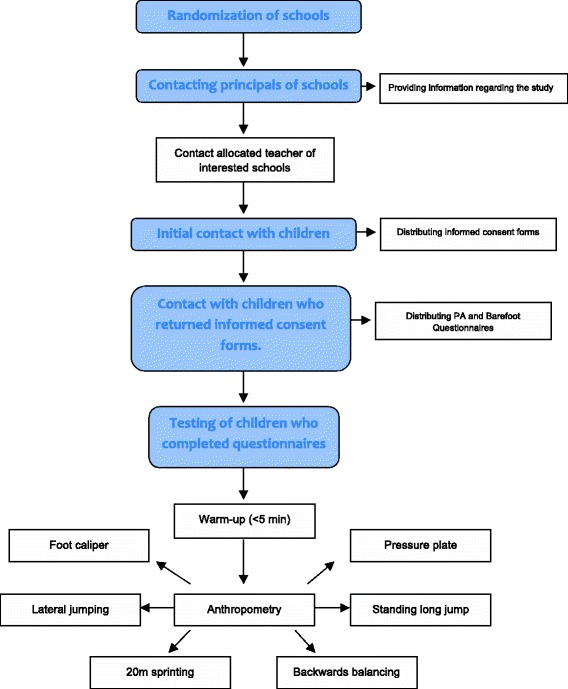
A flowchart demonstrating the participant flow through the study

After their first testing station, a participant will be transferred to the next vacant station without a fixed order. Testing station 1–3 will be completed only barefoot, while testing stations 4–7 will be performed in barefoot and shod conditions. The order of the condition is randomized a priori on the registration sheet which is distributed to the participants on the testing day. A flowchart displaying the participant flow through the study is shown in Fig. 1.

### Barefoot questionnaire

Participants will complete a six-item questionnaire used to decide if a child can be considered barefoot or shod. This questionnaire (Additional file 2) is developed specifically for this study and inquires if the child is barefoot on a three point Likert scale: most of the time, half of the time or barely/none of the time during 1) school 2) sports and 3) in and around the house. These questions will be asked twice: one for the warm weather and one for cold weather. For those in secondary school, questions are repeated for when they were in primary school. Due to the multi lingual culture of South Africa, the questions will be asked in Afrikaans, English and Xhosa. Children will be considered barefoot when they are always barefoot in and around the house and always barefoot in school *or* during sports -in warm weather- in primary school. In secondary school they have to have a similar level of barefootness during primary school *and* are always barefoot in and around the house currently.

### Outcomes

Prior to the start of the investigations in the schools, the German and South African research team will perform a joint training session over several days in Germany to ensure the identical use of the equipment and collection of data. Furthermore, the principal investigator (KH) will attend the first weeks of testing in South Africa to ensure accuracy in methodology and identical data collection. Inter-rater reliability testing will be performed to improve data quality and interpretability.

#### Foot mechanics

Foot mechanical outcomes will consist of dynamic arch index (dAI), static arch height index (sAHI) whilst sitting and standing (double limb support), foot and arch height pliability ratios, hallux angle, and footstrike pattern while jogging and running.

**Fig. 2:**
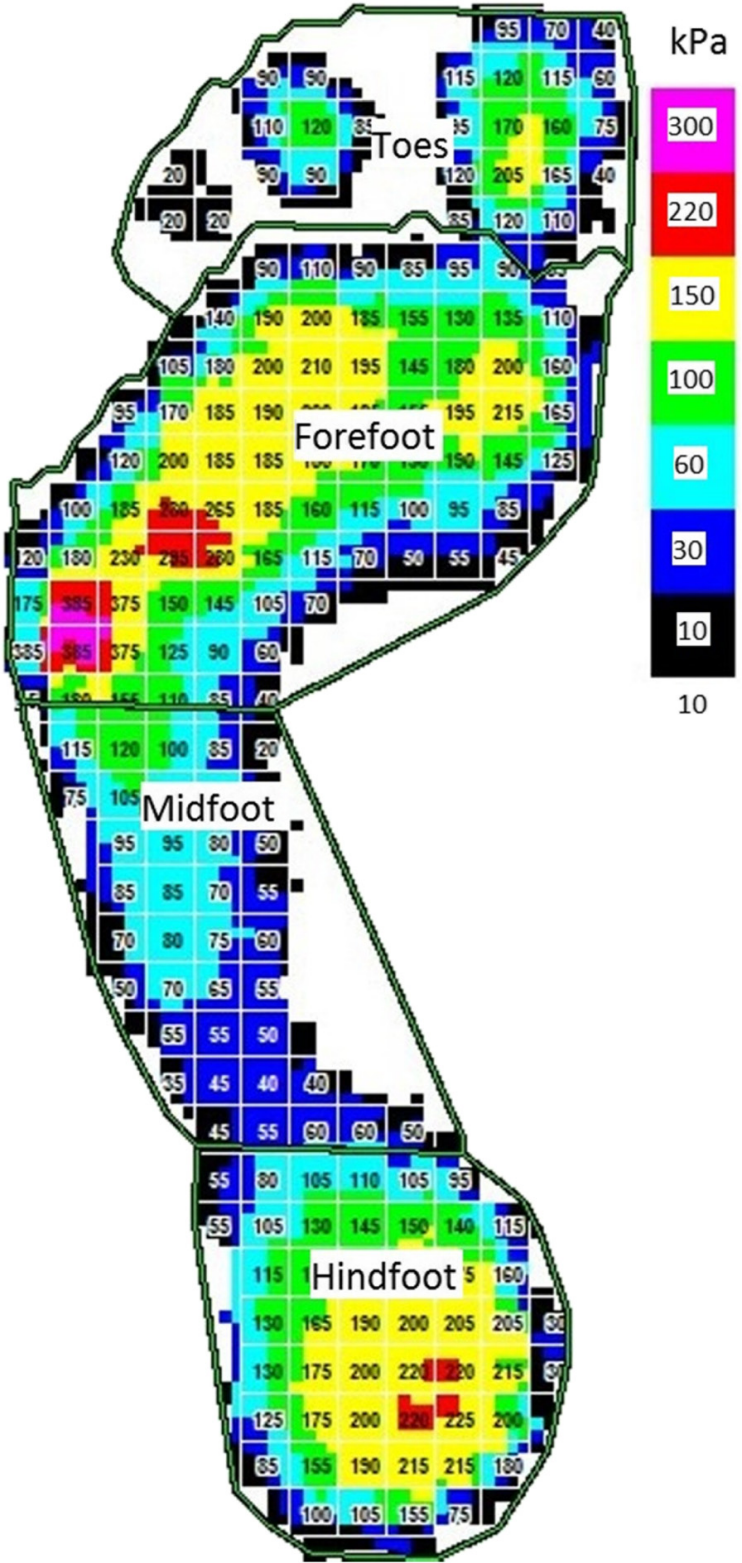
Example of a digital footprint with masking into forefoot, midfoot and hindfoot region for calculation of the dynamic arch index according to Cavanagh and Rodgers (1987)

The dAI describes the proportion of the middle third of a footprint compared to the whole footprint area (except for the toes) and was firstly described by Cavanagh and Rodgers [27] (Fig. 2). This method was shown to be valid and reliable in children [28, 29]. The dynamic footprints geometric will be acquired with a capacitance-based pressure platform system (Emed n50, Novel GmbH, Munich, Germany) using a two-step protocol [29, 30]. The platform has 6080 sensors in an area of 47,5 × 32 cm (4 sensors/cm^2^) and has been shown to be reliable in adult [31] and paediatric populations [29]. In order to level the platform to the ground, it will be embedded in a 300 cm wooden walkway.

Footprint data will be used to calculate the hallux angle according to R Donatelli and SL Wolf [32].

**Fig. 3:**
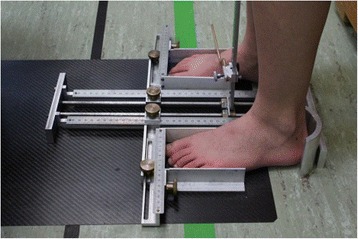
Heel to toe length, foot width and dorsum height measurements will be obtained using a specially constructed platform. Dorsum height will be measured at 50 % of the heel to toe length using the sliding caliper pictured on the right foot in this picture. Static arch height index will be defined as the ratio of dorsum height and heel to toe length

Measures of static foot anthropometrical data will be obtained with a specially constructed caliper (Fig. 3). This caliper consists of heel cups for the placement of both feet and sliding indicators for proper measurement of heel-to-toe length (HTL), foot width (FW) and dorsum height. HTL will be defined as the distance from the most posterior aspect of the foot to the most anterior part of the toes. Dorsum height will be measured at 50 % of HTL and the static arch height index (sAHI) will be defined as the ratio of dorsum height and HTL:

$$ Static\  arch\  height\  index = \frac{Dorsum\  height}{Heel-to- toe\  length} $$

Feet of the participants will be measured at sitting (10 % of body weight (BW)) and standing (50 % of BW) and the pliability ratio will be calculated according to Kadambande et al. [20]:

$$ Pliability\  ratio = \frac{HTL\ 50\%\  of\ BW \times FW\ 50\%\  of\ BW}{HTL\ 10\%\  of\ BW \times FW\ 10\%\  of\ BW} $$

Foot strike patterns will be captured during 20 m jogging and both sprinting trials in each condition at the 17.5 m mark using a wide-angle high speed camera (GoPro HD Hero 4, GoPro Inc., San Mateo, California, USA). The camera will be positioned 150 cm from the midline orthogonal to the marked running way and set to record 120 frames per second at a resolution of 1280 × 720 pixels. After testing, videos will be processed using a video editing software (Adobe Premiere Pro CS 6, Adobe Systems, San Jose, California, USA) and rated independently by two reviewers, with a third experienced reviewer for consensus. A rearfoot strike is defined as a first ground contact with the heel or the rear third of the foot, while a forefoot strike is present when the anterior part of the foot first contacts the ground. For a midfoot strike heel and anterior part of the foot contact the ground simultaneously. This method has been used successfully in other studies to determine the foot strike pattern [33].

#### Motor performance

Participants will complete multiple tests to assess motor performance; 20 m sprint [34], lateral jumping, standing long jump, and backwards balancing [35]. Each station will be performed in two conditions: barefoot and wearing sport shoes.

Preceding the lateral jumping (Additional file 3), participants will stand in one half of a square next to a line, indicated on the floor by masking tape. He/she will be instructed to jump sideways as fast as possible for 15 s. One minute rest will be given between the two trials per condition and the average score of each condition will be used for analysis. For the standing long jump (Additional file 4), a participant will be instructed to stand with their toes adjacent to a labelled start line, bend at the knees whilst swinging the arms backwards and subsequently, jump as far as possible on to a soft rebounding mat. One of the researchers will place a rod at the most posterior of where the participant landed and will read off the distance on the measuring tape attached parallel to the rebound mat on the floor. This will be repeated three times per condition and the distance of the best trial per condition is used for analysis. A sprinting lane of 20 m (Additional file 5) will be indicated on the floor by a start line labelled by tape and two pylons at 0, 10 and 20 m, as well as one meter after the 20 m mark as a dummy. A time sensor device will be placed at the start, 10 and 20 m. In Germany mobile magnetic timing gates from Humotion SmarTracks (Münster, Germany) will be used and Brower Timing Systems speed gates (Salt Lake City, UT, USA) in South Africa. The 10 m and 20 m time of the best trial per condition will be used for analysis. Lastly participants will be asked to walk backwards (Additional file 6) over a 6 cm, 4.5 cm and 3 cm wide balance beams with, respectively, 2 trials per beam [35]. The first step after the starting position will not be counted, every subsequent step will then be counted until one foot touches the ground or a maximum of 8 steps per beam is achieved. The children will be instructed to look up straight (with an X on eye level) and put their next step directly behind the other foot. The scores of two trials on each of the three beams will be added to a total score per condition for analysis (max 48 points).

### Data collection and management

Data will primarily be collected on paper sheets and then transferred to electronic spreadsheets at each testing site (Germany and South Africa). The entered data will be checked by the principal investigators. The pedobarographic data will be collected within the provided software (Novel database pro m, Version 24.3.20, Novel GmbH, Munich, Germany) and then exported as ASCII files and included into the spreadsheet. Electronic data will not include any confidential participant information and will then be transferred via secure servers to Germany for further processing and statistical analysis. As specified in the ethically approved data protection declaration for the participants, the encoded data will be stored on external hard disks in a locked safety cabinet for 10 years. Participants have the right to obtain the personalised data. Data analysis and publication will only be done in an anonymised format.

### Sample size

Although we did not opt to distinguish primary or secondary outcome measures, the dAI was used to enable a sample size calculation. Muller et al. [36] has shown that the average dynamic arch index (μ: 0,19) and the accompanying standard deviation (SD: 0065) are stable between the ages of 6 and 13. The minimal important difference was set at 20 % of the average (0.19; i.e., 0.038). With a two-sided significance level of 0.05, and assuming a power of 0.8, a minimum of 16 participants per age group, per country, had to be included for this study. Due to the diversity of the study, additional power calculations have been performed. Based on the arch height ratio derived from Waseda et al. [22] the sample size for arch height ratio (navicular height*100/foot length) should be *n* = 12 per age group (μ = 15,0; SD = 2,6). Furthermore, based on a preliminary study on children, the sample size based on the standing long jump should be *n* = 18 (μ = 152,9; SD = 31,5), for lateral jumping *n* = 22 (μ = 38; SD = 9,2) and for 20 m sprint *n* = 12 (μ = 3,81; SD = 0,64). Therefore, our *n* = 20 per group seems to be sufficient. In order to allow for these differences and other unknown variances within the other variables we choose to increase the number from 16 to 20 participants per age group, per country.

### Statistical analysis

Descriptive data will be presented using descriptive statistics. The participant’s barefoot questionnaire and the PAQ outcomes will be compared to all attending children in 2 primary (1 rural, 1 urban) and 2 secondary schools to assess external validity. The outcome measures will be evaluated for normality using Shapiro-Wilkins and visually using P-P plots, if possible, non-normal distributed data will be adjusted. Mixed Models linear regression will be used to assess if the foot mechanical and motor performance outcomes differ between the barefoot and the shod participants (fixed factors). Furthermore, we will test differences due to being barefoot or shod change by age by adding an interaction of age*group to the linear regression. Differences in foot strike pattern during jogging and sprinting between barefoot and shod participants will be assessed using ordinal regression. The school of a participant will be added to the model as a random effect in order to adjust for possible differences between the schools and their geographical location. Furthermore, Gender, BMI, PAQ-Score, ethnicity and inside/outside testing will be tested for confounding in all models (regression coefficient changes >10 %). We hypothesize that older boys could have higher arches (lower dAI) [37] and perform better on motor performance tasks, while girls’ feet will show a higher pliability. BMI increases the dAI and possibly decreases motor performance [38, 39]. A higher level of physical activity (higher PAQ score) is probably related to better motor performance. And lastly, Caucasians could have higher arches (lower dAI) [40].

We anticipate that gender might modify the effect of being barefoot or shod on the outcome measures and therefore gender will additionally tested as an effect modifier (interaction between outcome and gender *p* < 0.1). In all cases, a significance level of 5 % is pre-stipulated.

## Discussion

The study as described in this paper will aim to evaluate the influence of being habitually barefoot or shod on foot mechanics and motor performance of children and adolescents aged 6–18 years. Additionally we will test whether differences between habitually barefoot and shod children are age-dependent.

### Barefoot questionnaire

In designing the study, decisions have been made that could influence the outcome and its interpretations. The level of barefootness of the participants in South Africa is one of those decisions since the current literature descriptions are diverse and even ambiguous at times.

Some studies use a percentage of yearly mileage [8, 9] or running time [41] to define a habitually barefoot person. Other studies define a habitually barefoot individual as being barefoot for all life [8, 14, 16, 20] or as living in an area where it is common to be barefoot [15, 19, 21]. Children in Germany are shod most of the time due to the climate and the culture of wearing shoes when outside. In South Africa a culture exists for children of being barefoot outside, and for younger children, even during sports and at school. Furthermore, the warmer climate allows the children to do so for the biggest part of the year. To distinguish between children who are habitually barefoot, a barefoot questionnaire has been developed. Participants in primary school (age range 6–12 years) are defined as habitually barefoot when they are always barefoot in and around the house as well as at school or during sports when it is warm. In secondary school being habitually barefoot is defined as always being barefoot in and around the house and having had the level of barefootness at primary school as previously described. The distinction between primary and secondary school was made because all secondary schools require wearing a uniform that includes wearing shoes and participation in most sports do as well. Therefore, it wasn’t feasible to include participants after the age of 13 years who had a similar level of barefootness as their counterparts in primary school.

Similar differences in the level of barefootness are found in other studies. For instance, UB Rao and B Joseph [15] compared barefoot children to shod children in India where the latter group mainly wore flip-flops or shoes with a soft upper. One could argue that the influence of these ‘shoes’ is not comparable to wearing hard soled shoes with a rigid upper. Other studies compared participants who were shod or barefoot all of the time, but those participants were from different ethnicities [14, 16, 20]. Our study will aim to recruit participants in South Africa from similar ethnic backgrounds to the German children. This will allow for increased comparability of the independent variable: being habitually barefoot or shod. However, the diverse ethnic backgrounds of the South African population will not allow for an exact similar recruitment compared to the German population. We will adjust for this discrepancy by adding ethnicity as a confounding variable to the statistical evaluations. And lastly, even though we think that the use of a barefoot questionnaire is preferable compared to the mentioned studies in assessing the level of barefootness, a limitation of the use of the questionnaire is that it will not be validated a priori.

### Foot mechanics

A major purpose of this study is the assessment of the foot mechanics. Differences in foot anthropometrics have been found between habitually barefoot and shod individuals [8, 14, 16]. Special focus will be laid on the medial longitudinal arch and its development over age. Most studies report that the development of the arch mainly occurs until the age of 6–8 years [42], while other studies state that substantial changes to the arch morphology can still occur during adolescence [43]. Intrinsic and extrinsic (e.g., footwear) factors influence the development of the foot [42] and thus the habitual footwear use will be examined.

The classification and measurement of foot mechanics is still a controversial topic [44, 45]. Indirect (footprints analogue or digital pedobarographic) and direct methods (clinical assessment, caliper, radiographs) exist with certain strengths and limitations within each method. In this study we will assess the foot mechanics using a dynamic indirect (pedobarographic) and a static direct method (caliper). The pedobarographic measurement of the arch index has been shown to be a simple and reproducible method [46] that show higher reliability than navicular height measurement in pediatric populations [47]. Nonetheless, there is variability in the dynamic arch index and we will address this problem by including three valid trials per leg in each participant using a two-step protocol that has been shown to be reliable for children [29, 48, 49]. Invalid trials will be defined by the trained investigator when participants target the platform, step on the border or alter their gait for example due to distraction by the other children. In that case the trial will be excluded and re-measured. Using digital pedobarographic measures and the derived dAI is a proxy for the longitudinal medial arch. It has been hypothesized that dAI might be influenced by other variables than the skeletal arch itself, for instance the amount of adipose tissue [38]. A radiographic measure (X-ray) of the arch would be a more valid measure. But besides the exposure to radiation, this method is not feasible while testing in the field with the current sample size of the study. Thus, the non-invasive static and dynamic arch assessment used in this study will be preferred. By statistically adjusting for BMI, we aim to increase validity of the dAI measure.

The relationship between static arch height index and dynamic arch index has been shown for adults [30] and there is a high reliability for dorsum height when normalized to foot length at 50 % of weight bearing [50]. Due to limitations of each method, a strength of this study is the use of two different tools for foot assessments. The clinical relevance of the findings still has to be elucidated.

Assessing the foot pliability within this study shall help to understand the effect of habitual footwear use on the flexibility and mobility of the foot. The foot consists of 26 bones and 33 joints of which most are actively articulated. There is evidence that footwear diminishes the pliability of the foot that could facilitate pathologies like the hallux valgus, hallux rigidus and pes planus [20, 51].

The visual determination of the foot strike pattern has been used in other studies [8]. It is not as exact as the biomechanical determination [52] but is practical for the assessment of a large cohort [33]. Limitations of previous studies on the effect of barefoot walking or running on foot mechanics include a limit of outcome measures and therefore a limited validity. In our study, we will aim to increase validity by including different variables (dAI, arch height pliability, hallux angle and foot strike pattern) that measure different aspects of foot mechanics.

### Motor performance

The criterion for the selection of these motor performance tests is related to the expected effects of habitual barefoot locomotion on lower limb gross motor skills, even though the evidence for the effects of habitual barefoot locomotion on motor performance is unclear [Hollander K, Heidt C, Van der Zwaard B, Braumann KM, Zech A. Long-term effects of habitual barefoot running and walking: a systematic review. Manuscript submitted for publication].

The tests used to assess motor performance have been used in other studies, and have been shown to be valid and reliable [34, 35]. Even though all tests have been extensively practiced by the main researchers, the researchers from both countries combined and separate, it is imaginable that regional characteristics and the use of different examiners as well as test equipment will influence the results. An example is that the sprinting will be mostly done outdoors in South Africa due to a lack of indoor sport facilities at some schools which might be influential on the 10 m and 20 m sprint time. By adding a variable for outdoor/indoor running we will test if it acts as a confounder and adjust the outcome accordingly. Another difference between testing in Germany and South Africa is the speed gates used. Mobile magnetic timing gates will be used in Germany while in South Africa single beam speed gates will be used [53]. The latter is less accurate, however, the measurement error is still small (0.01 s) [54]. Therefore, no adjustments will be made when comparing the two countries.

Another limitation could be that measuring the distance during the standing long jump is done visually by one of the researchers by placing a right-angled rod at the place the heel landed. By using the average of three tries the influence of possible random measurement errors would decrease. Nonetheless, to provide comparability to other studies [34, 35], we will use the best of three tries. When using the backward balance test, a possible limitation would be a ceiling effect since there is a maximum of 8 steps per beam. However, by adding the steps for all the beams and the likelihood of not reaching the maximum 8 steps on the 4.5 and 3 cm beam, it is unlikely that a ceiling effect will occur. The score (i.e., the sum of the trials) will be treated as a continues variable during data analysis.

The important strength of this study will be twofold. First of all, we will try to establish an exact definition of “habitual barefootness” using a barefoot questionnaire. Secondly, we will compare two large cohorts with different footwear habits to determine the influence of being habitually barefoot on foot mechanics and motor performance of children and adolescents aged 6–18 years. Therefore, the results will contribute to the better understanding of the long-term effects of barefoot locomotion on foot mechanics and motor performance.

## Additional files

Additional file 1: Information, consent and assent forms in different languages. (PDF 4314 kb) Additional file 2: Barefoot questionnaire for primary and secondary school. (PDF 67 kb) Additional file 3: Participant at the lateral jumping station. (PDF 175 kb) Additional file 4: Participant at the standing long jump station. (PDF 175 kb) Additional file 5: Participant at the 20 m sprinting station. (PDF 134 kb) Additional file 6: Participant at the backwards balancing station. (PDF 183 kb)

## Abbreviations

Barefoot LIFE: Barefoot Locomotion for Individual Foot- and health Enhancement
BMI: Body mass index
BW: Body weight
dAI: Dynamic arch index
FW: Foot width
HTL: Heel-to-toe length
PAQ-A: Physical activity questionnaire for adolescent
PAQ-C: Physical activity questionnaire for children
sAHI: Static arch height index
SD: Standard deviation

## References

[1] Perkins KP, Hanney WJ, Rothschild CE (2014). The risks and benefits of running barefoot or in minimalist shoes: a systematic review. Sports Health.

[2] Tam N, Wilson JLA, Noakes TD, Tucker R (2014). Barefoot running: an evaluation of current hypothesis, future research and clinical applications. Br J Sports Med.

[3] Hall JPL, Barton C, Jones PR, Morrissey D (2013). The biomechanical differences between barefoot and shod distance running: a systematic review and preliminary meta-analysis. Sports Med (Auckland, NZ).

[4] Franklin S, Grey MJ, Heneghan N, Bowen L, Li FX (2015). Barefoot vs common footwear: a systematic review of the kinematic, kinetic and muscle activity differences during walking. Gait Posture.

[5] Fuller JT, Bellenger CR, Thewlis D, Tsiros MD, Buckley JD (2015). The effect of footwear on running performance and running economy in distance runners. Sports Med.

[6] Wegener C, Hunt AE, Vanwanseele B, Burns J, Smith RM (2011). Effect of children’s shoes on gait: a systematic review and meta-analysis. J Foot Ankle Res.

[7] Hollander K, Argubi-Wollesen A, Reer R, Zech A (2015). Comparison of minimalist footwear strategies for simulating barefoot running: a randomized crossover study. PLoS One.

[8] Lieberman DE, Venkadesan M, Werbel WA, Daoud AI, D’Andrea S, Davis IS, Mang’Eni RO, Pitsiladis Y (2010). Foot strike patterns and collision forces in habitually barefoot versus shod runners. Nature.

[9] Altman AR, Davis IS (2016). Prospective comparison of running injuries between shod and barefoot runners. Br J Sports Med.

[10] Tam N, Tucker R, Astephen Wilson JL (2016). Individual responses to a barefoot running program: insight into risk of injury. Am J Sports Med.

[11] Warne JP, Moran KA, Warrington GD (2015). Eight weeks gait retraining in minimalist footwear has no effect on running economy. Hum Mov Sci.

[12] Warne JP, Warrington GD (2014). Four-week habituation to simulated barefoot running improves running economy when compared with shod running. Scand J Med Sci Sports.

[13] Arulsingh W, Pai G (2015). A study of foot defects, deformities and diseases among shod and barefoot middle and long distance runners - cross sectional study. Int J Curr Res Rev.

[14] Echarri JJ, Forriol F (2003). The development in footprint morphology in 1851 Congolese children from urban and rural areas, and the relationship between this and wearing shoes. J Pediatr Orthop B.

[15] Rao UB, Joseph B (1992). The influence of footwear on the prevalence of flat foot. A survey of 2300 children. J Bone Joint Surg Br.

[16] D’AoÛt K, Pataky TC, De Clercq D, Aerts P (2009). The effects of habitual footwear use: foot shape and function in native barefoot walkers. Footwear Sci.

[17] Ashizawa KK C, Kusumoto A, Narasaki S (1997). Relative foot size and shape to general body size in Javanese, Filipinas and Japanese with special reference to habitual footwear types. Ann Hum Biol.

[18] Shu Y, Mei Q, Fernandez J, Li Z, Feng N, Gu Y (2015). Foot morphological difference between habitually shod and unshod runners. PLoS One.

[19] Sim-Fook L, Hodgson A (1958). A comparison of foot forms among the non-shoe and shoe-wearing Chinese population. J Bone Joint Surg.

[20] Kadambande S, Khurana A, Debnath U, Bansal M, Hariharan K (2006). Comparative anthropometric analysis of shod and unshod feet. Foot.

[21] Barnicot NA, Hardy RH (1955). The position of the hallux in West Africans. J Anat.

[22] Waseda A, Suda Y, Inokuchi S, Nishiwaki Y, Toyama Y (2014). Standard growth of the foot arch in childhood and adolescence--derived from the measurement results of 10,155 children. Foot Ankle Surg.

[23] Castro-Aragon O, Vallurupalli S, Warner M, Panchbhavi V, Trevino S (2009). Ethnic radiographic foot differences. Foot Ankle Int.

[24] Chan AW, Tetzlaff JM, Gotzsche PC, Altman DG, Mann H, Berlin JA, Dickersin K, Hrobjartsson A, Schulz KF, Parulekar WR (2013). SPIRIT 2013 explanation and elaboration: guidance for protocols of clinical trials. BMJ.

[25] Crocker PR, Bailey DA, Faulkner RA, Kowalski KC, McGrath R (1997). Measuring general levels of physical activity: preliminary evidence for the Physical Activity Questionnaire for Older Children. Med Sci Sports Exerc.

[26] Kowalski K, Crocker P, Donen R. The Physical Activity Questionnaire for Older Children (PAQ-C) and Adolescents (PAQ-A) Manual. Saskatoon: College of Kinesiology, University of Saskatchewan; 2004.

[27] Cavanagh PR, Rodgers MM (1987). The arch index: a useful measure from footprints. J Biomech.

[28] Cousins SD, Morrison SC, Drechsler WI (2012). The reliability of plantar pressure assessment during barefoot level walking in children aged 7–11 years. J Foot Ankle Res.

[29] Tong JW, Kong PW (2013). Reliability of footprint geometric and plantar loading measurements in children using the Emed((R)) M system. Gait Posture.

[30] Teyhen DS, Stoltenberg BE, Collinsworth KM, Giesel CL, Williams DG, Kardouni CH, Molloy JM, Goffar SL, Christie DS, McPoil T (2009). Dynamic plantar pressure parameters associated with static arch height index during gait. Clin Biomech.

[31] Putti AB, Arnold GP, Cochrane LA, Abboud RJ (2008). Normal pressure values and repeatability of the Emed ST4 system. Gait Posture.

[32] Donatelli R, Wolf SL (1996). The Biomechanics of the foot and ankle.

[33] Hasegawa H, Yamauchi T, Kraemer WJ (2007). Foot strike patterns of runners at the 15-km point during an elite-level half marathon. J Strength Cond Res.

[34] Bös K, Schlenker L, Büsch D, Lämmle L, Müller H, Oberger J, Seidel I, Tittlbach S (2009). Deutscher Motorik-Test 6–18 (DMT 6–18).

[35] Woll A, Kurth BM, Opper E, Worth A, Bos K (2011). The ‘Motorik-Modul’ (MoMo): physical fitness and physical activity in German children and adolescents. Eur J Pediatr.

[36] Muller S, Carlsohn A, Muller J, Baur H, Mayer F (2012). Static and dynamic foot characteristics in children aged 1–13 years: a cross-sectional study. Gait Posture.

[37] Saghazadeh M, Kitano N, Okura T (2015). Gender differences of foot characteristics in older Japanese adults using a 3D foot scanner. J Foot Ankle Res.

[38] Wearing SC, Hills AP, Byrne NM, Hennig EM, McDonald M (2004). The arch index: a measure of flat or fat feet?. Foot Ankle Int.

[39] Mueller S, Carlsohn A, Mueller J, Baur H, Mayer F (2016). Influence of obesity on foot loading characteristics in gait for children aged 1 to 12 years. PLoS One.

[40] Stolwijk NM, Duysens J, Louwerens JW, van de Ven YH, Keijsers NL (2013). Flat feet, happy feet? Comparison of the dynamic plantar pressure distribution and static medial foot geometry between Malawian and Dutch adults. PLoS One.

[41] Goss DL, Gross MT. Relationships among self-reported shoe type, footstrike pattern, and injury incidence. US Army Med Dep J. 2012;25–30. 23007933

[42] Staheli LT (1991). Shoes for children: a review. Pediatrics.

[43] Stavlas P, Grivas TB, Michas C, Vasiliadis E, Polyzois V (2005). The evolution of foot morphology in children between 6 and 17 years of age: a cross-sectional study based on footprints in a Mediterranean population. J Foot Ankle Surg.

[44] Jonely H, Brismee JM, Sizer PS, James CR (2011). Relationships between clinical measures of static foot posture and plantar pressure during static standing and walking. Clin Biomech.

[45] Mootanah R, Song J, Lenhoff MW, Hafer JF, Backus SI, Gagnon D, Deland JT, Hillstrom HJ (2013). Foot Type Biomechanics Part 2: are structure and anthropometrics related to function?. Gait Posture.

[46] Yalcin N, Esen E, Kanatli U, Yetkin H (2010). Evaluation of the medial longitudinal arch: a comparison between the dynamic plantar pressure measurement system and radiographic analysis. Acta Orthop Traumatol Turc.

[47] Gilmour JC, Burns Y (2001). The measurement of the medial longitudinal arch in children. Foot Ankle Int.

[48] Oladeji O, Stackhouse C, Gracely E, Orlin M (2008). Comparison of the two-step and midgait methods of plantar pressure measurement in children. J Am Podiatr Med Assoc.

[49] Hollander K, Scholz T, Braumann KM, Zech A (2016). Correlation between static and dynamic foot arch measurement in children – Preliminary study of the Barefoot LIFE project. Gait Posture.

[50] Saltzman CL, Nawoczenski DA, Talbot KD (1995). Measurement of the medial longitudinal arch. Arch Phys Med Rehabil.

[51] Perera AM, Mason L, Stephens MM (2011). The pathogenesis of hallux valgus. J Bone Joint Surg Am.

[52] Hollander K, Riebe D, Campe S, Braumann K-M, Zech A (2014). Effects of footwear on treadmill running biomechanics in preadolescent children. Gait Posture.

[53] Earp JE, Newton RU (2012). Advances in electronic timing systems: considerations for selecting an appropriate timing system. J Strength Cond Res.

[54] Hammami R, Makhlouf I, Chtara M, Padulo J, Chaouachi A (2014). The contribution of vertical explosive strength to sprint performance in children. Sport Sci Health.

