# Supplementary material for: The effects of being habitually barefoot on foot mechanics and motor performance in children and adolescents aged 6–18 years: study protocol for a multicenter cross-sectional study (Barefoot LIFE project)
Source: J Foot Ankle Res. 2016 Sep 2;9(1):36. doi: 10.1186/s13047-016-0166-1 (PMC5010736; doi:10.1186/s13047-016-0166-1)
Supplement: Additional file 1: — Information, consent and assent forms in different languages. (PDF 4314 kb) [file 13047_2016_166_MOESM1_ESM.pdf]

STELLENBOSCH UNIVERSITY

PARTICIPANT INFORMATION LEAFLET AND ASSENT FORM

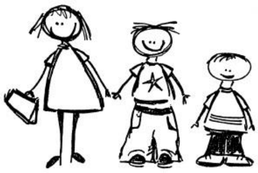

TITLE OF THE RESEARCH PROJECT:
Moving Feet – A Study where we compare school-aged children who normally walks barefoot to those who normally wear shoes.

RESEARCHER'S NAME: Elbé de Villiers

ADDRESS: Department of Sport Science, Stellenbosch University

CONTACT NUMBER: 021 808 4735 / 021 808 4735

What is RESEARCH*?*
Research is something we do to find **NEW KNOWLEDGE** about the way things (and people) work. We use research projects or studies to help us find out more about children and teenagers and the things that affect their lives, their schools, their families and their health. We do this to try and make the world a better place!

What is this research project all about?
During this project we want to see what effect your everyday shoes have on:
The way you walk
The shape of your feet
Your balance
The distance that you can jump

Why have I been invited to take part in this research project?
You were invited because you are a pupil in one of the schools that was chosen for the study. You are healthy, do not have an injury and you are the right age.

Who is doing the research?
My name is Elbé de Villiers. I am a Biokineticist working at Stellenbosch University. My job is to help people get better after they had an injury, where in an accident or where very ill. We help them by doing specific exercises.

What will happen to me in this study?
During the study we will do a few tests.
First of all we will measure your height and weight.
Then we will do a warm-up (light jogging and stretches) to get you ready for the other tests.
We will ask you to walk a few metres over a platform. We will take measurements of your foot while you are standing and sitting
The balance test is next. You will need to walk backwards on three different sized plank, 3 times.
You will be asked to jump forward as far as you can 3 times and jump sideways as many times as possible in 15 seconds. You will do this twice.
Next you will jog and run 20 metres while being recorded by a video camera. We want to see how you put your foot down while running. Only the running will be done twice and the time it takes you to complete this will be taken.
Lastly we will measure your hand grip strength.

Can anything bad happen to me?
Nothing bad can happen to you during the study. You will only run short distances and jump three times. The only thing that might happen is that your muscles might feel uncomfortable.
We will show you how to do everything.

Will anyone know I am in the study?
Nobody have to know that you are part of the study. Your specific results will only be known by Elbé.

Who can I talk to about the study?
If you have questions or want to speak to someone about the study you can contact: Elbé de Villiers (cell phone: 084 515 7642; email: edup@sun.ac.za) or Dr Ranel Venter (cell phone: 083 309 2894; email: rev@sun.ac.za).

What if I do not want to do this?
No one can force you to be part of the study. If you do not want to do this, you do not have to. Even if your parents allowed you and signed the form, you still do not have to do it.
If you said that you want to be part of the study and decide later on that you do not want to do it any more, nothing will happen to you and you can just stop being part of it.

Do you understand this research study and are you willing to take part in it?

YES NO

Has the researcher answered all your questions?

YES NO

Do you understand that you can STOP being in the study at any time?

YES NO

___________________________ ______________________

Signature of Child Date

UNIVERSITEIT•STELLENBOSCH•UNIVERSITY
jou kennisvennoot • your knowledge partner

STELLENBOSCH UNIVERSITY
CONSENT TO PARTICIPATE IN RESEARCH

Moving feet – a comparative study of school children who normally wear shoes and those who normally walk barefoot

I am Elbé de Villiers (a PhD student in Sport Science) of the Department of Sport Science at Stellenbosch University. I would like to invite your child to participate in my research study. The results of the study will form part of the thesis for my doctoral degree in Sport Science. Your child has been chosen as a possible participant in the study because he/she is in one of the participant schools and also is of the right age.

1. PURPOSE OF THE STUDY

The main purpose of this study is to determine the effect that shoes have on the developing foot. I will also determine whether shoes influence children's ability to move.

2. PROCEDURES

If you agree that your child may take part in this study, your child will have to undergo the following tests and measurements:

Anthropometric measurement: Your child's length and weight will be measured.

Complete a questionnaire on physical activity: This is done to determine how active your child is.

Jogging and running for 20 metres: While your child runs, he/she will be recorded on a video camera. The child will be asked to do this three times with and without shoes. The video is just to determine how your child lands with his feet while running.

Balance tests: Your child will be asked to walk backwards on three different sized bars. This will be done twice on each bar with and without shoes.

Jumps: Your child will be asked to jump as far as he/she can with both feet together. The distance will be measured. Your child will do this jump three times with and without shoes.
Next your child will be asked to jump sideways as many times as possible in 15 seconds. They will do it twice with and without shoes.

Foot shape: Your child will be asked to walk over a platform with a pressure plate embedded in it. They will also have to stand on a foot measuring platform, which then will determine the child's foot length and breadth as well as the height of his/her foot bridge while standing and seated.

Grip strength: Your child's grip strength will be determined by using a hand grip calliper.

3. POTENTIAL RISKS AND DISCOMFORT

Although some of the tests might be unknown to your child, they are simple tests. They should not make your child exceptionally tired or cause any discomfort.

4. POTENTIAL BENEFITS FOR STUDY PARTICIPANTS AND/OR SOCIETY

Your child will gain no direct benefit from the study.

The study does hold benefits for knowledge in the field of sport science, however, and specifically on the effect of shoes on children's feet and their ability to move. The results could possibly also provide shoe manufacturers with the necessary knowledge in the future to design shoes that are beneficial for the development of children's feet.

5. REMUNERATION FOR PARTICIPATION

Your child will not be paid for participation in this study.

6. CONFIDENTIALITY

Any information that is obtained in connection with this study and that could reveal your child's identity will remain confidential and will only be revealed with your consent or if required by law. Confidentiality will be maintained by storing the data on a personal computer with a password. Only the researcher and the supervisor will be able to look at the data. The data will be dealt with anonymously at all times.

If the research should be published, the data will be discussed in general – in other words for the group as a whole.

7. PARTICIPATION AND WITHDRAWAL

You can decide whether or not your child may participate in this study. If you offer that your child may participate, you may still withdraw him/her from the study at any stage without this holding any negative consequences for your child. The researcher could also decide to remove your child from the study should circumstances require this.

8. DETAILS OF RESEARCHERS

If you have any questions on the research or if anything about it bothers you, you are welcome to contact us:

Elbé de Villiers (cell phone 084 515 7642; e-mail edup@sun.ac.za) or Dr Ranel Venter (cell phone 083 309 2894; e-mail rev@sun.ac.za)

9. RIGHTS OF RESEARCH PARTICIPANTS

You may withdraw your consent at any stage and discontinue your child's participation, without any negative consequences. Your child will not waive any legal claims or rights by taking part in this research study. For any questions about your child's rights as a study participant, contact Ms Maléne Fouché at the Stellenbosch University Division for Research Development [mfouche@sun.ac.za; 021 808 4622].

SIGNATURE OF PARENT / GUARDIAN

I was given a copy of the letter with information.
I was given the opportunity to ask questions, and they were answered satisfactorily.

I consent that ________________________________ may participate in this study. I have received a copy of this form.

____

Name of parent/guardian

___________________________________ ________________

Signature of parent/guardian **Date**

Physical Address:

Street number and name:____

Area / Suburb: ____

Town / City: ____

UNIVERSITEIT•STELLENBOSCH•UNIVERSITY
jou kennisvennoot • your knowledge partner

STELLENBOSCH UNIVERSITY
CONSENT TO PARTICIPATE IN RESEARCH

Moving feet – a comparative study of school children who normally wear shoes and those who normally walk barefoot

I am Elbé de Villiers (a PhD student in Sport Science) of the Department of Sport Science at Stellenbosch University. I would like to invite you to participate in my research study. The results of the study will form part of the thesis for my doctoral degree in Sport Science. You have been chosen as a possible participant in the study because you are in one of the participant schools and also are of the right age.

1. PURPOSE OF THE STUDY

The main purpose of this study is to determine the effect that shoes have on the developing foot. I will also determine whether shoes influence children's ability to move.

2. PROCEDURES

If you agree to take part in this study, you will have to undergo the following tests and measurements:

Anthropometric measurement: Your length and weight will be measured.

Complete a questionnaire on physical activity: This is done to determine how active you are.

Questionnaire on being barefoot: This will be done to determine how often you are barefoot.

Jogging and running for 20 metres: First you will jog and then sprint for 20 metres. While doing this, you will be recorded on a video camera. You will be asked to do the sprinting twice with and without shoes. The video is just to determine how you land with your feet while running.

Balance tests: You will be asked to walk backwards on three different sized bars. This will be done twice on each bar with and without shoes.

Jumping: You will be asked to jump as far as you can with both feet together. The distance will be measured. You will do this jump three times with and without shoes.
With the next jump, you will have to jump sideways as many times as possible in 15 seconds. The jumps will be counted and you will do it twice with and without shoes.

Foot shape: You will be asked to walk over a platform with a pressure plate embedded in it.
You would also have to stand with both legs on a foot measuring platform and your arch height, foot length and foot width will be measured by a calliper while you are standing and being seated.

Grip strength: Your grip strength will be determined by using a hand grip calliper.

3. POTENTIAL RISKS AND DISCOMFORT

Although some of the tests might be unknown to you, they are simple tests. They should not make you exceptionally tired or cause any discomfort.

4. POTENTIAL BENEFITS FOR STUDY PARTICIPANTS AND/OR SOCIETY

You will gain no direct benefit from the study.

The study does hold benefits for knowledge in the field of sport science, however, and specifically on the effect of shoes on children's feet and their ability to move. The results could possibly also provide shoe manufacturers with the necessary knowledge in the future to design shoes that are beneficial for the development of children's feet.

5. REMUNERATION FOR PARTICIPATION

You will not be paid for participation in this study.

6. CONFIDENTIALITY

Any information that is obtained in connection with this study and that could reveal your identity will remain confidential and will only be revealed with your consent or if required by law. Confidentiality will be maintained by storing the data on a personal computer with a password. Only the researcher and the supervisor will be able to look at the data. The data will be dealt with anonymously at all times.

If the research should be published, the data will be discussed in general – in other words for the group as a whole.

7. PARTICIPATION AND WITHDRAWAL

You can decide whether or not you want to participate in this study. If you offer that you will participate, you may still withdraw from the study at any stage without this holding any negative consequences for you. The researcher could also decide to remove you from the study should circumstances require this.

8. DETAILS OF RESEARCHERS

If you have any questions on the research or if anything about it bothers you, you are welcome to contact us:

Elbé de Villiers (cell phone 084 515 7642; e-mail edup@sun.ac.za) or Dr Ranel Venter (cell phone 083 309 2894; e-mail rev@sun.ac.za)

9. RIGHTS OF RESEARCH PARTICIPANTS

You may withdraw your consent at any stage and discontinue your participation, without any negative consequences. You will not waive any legal claims or rights by taking part in this research study. For any questions about your rights as a study participant, contact Ms Maléne Fouché at the Stellenbosch University Division for Research Development [mfouche@sun.ac.za; 021 808 4622].

SIGNATURE OF PARTICIPANT

I was given a copy of the letter with information.
I was given the opportunity to ask questions, and they were answered satisfactorily.

I consent that I, _________________________________ will participate in this study. I have received a copy of this form.

____
Name of participant

_____________________________________ ________________
Signature of participant **Date**

IYUNIVESITHI YASESTELLENBOSCH

INCWADANA YEENKCUKACHA ZOMTHATHI-NXAXHEBA NEFOMU YEMVUME

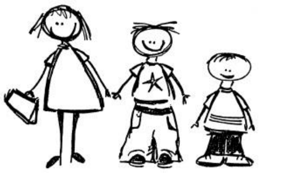

ISIHLOKO SEPROJEKTHI YOPHANDO:
Iintshukumo zeenyawo – uphando lokuthelekisa abantwana abathanda ukunxiba izihlangu kunye nabo bathanda ukuhamba ngeenyawo.

IGAMA LOMPHANDI: Elbé de Villiers

IDILESI: Isebe loBunzululwazi bezeMidlalo, kwiYunivesithi yaseStellenbosch

INOMBOLO YOQHAGAMSHELWANO: 021,808 (4735)21 938 4735

Luyintoni uPHANDO?
Uphando yinto esiyenzayo ukukhangela **ULWAZI OLUTSHA** malunga nendlela ezisebenza ngayo izinto (nabantu). Senza iiprojekthi zophando ukufumanisa ngakumbi ngabantwana abancinci kunye nabo sebekhulile kunye nezinto ezichaphazela ubomi babo, izikolo zabo, iintsapho zabo nempilo yabo. Sikwenza oku ukuze senze ihlabathi libe yindawo engcono!

Le projekthi yophando imalunga nantoni?
Ngexesha lokwenza le projekthi sifuna ukubona ukuba izihlangu zakho zemihla ngemihla ezi zinaliphi ifuthe ezinyaweni zakho:
Indlela ohamba ngayo
Ubume beenyawo zakho
Indlela obhalansa ngayo
Umgama onokuxhumela kuwo
Ixesha olithathayo ukubaleka umgama omfutshane nokujika uphinde ubuye umva

Bekutheni ukuze ndicelwe ukuba ndithathe inxaxheba kule projekthi yophando?
Ucelwe kuba ungumfundi kwesinye sezikolo esikhethelwe ukuba kwenziwe kuso uphando. Uphilile, awenzakalanga yaye ubudala bakho bobu sifunayo.

Lwenziwa ngubani olu phando?
Igama lam nguElbé de Villiers. Ndingumoluli womzimba osebenza kwiYunivesithi yaseStellenbosch. Umsebenzi wam kukunceda ukuba abantu babe ngcono emva kokufumana umenzakalo, naxa bebesengozini okanye xa bebegula kakhulu. Sibanceda ngokuthi sibenzise imithambo ethile.

Kuza kwenzeka ntoni kum kolu phando?
Kolu phando siza kwenza iimvavanyo ezimbalwa.
Okokuqala siza kumeta ubude bakho nobunzima bakho.
Emva koko siza kwenza imithambo yokuzilungiselela (ukubaleka kancinci nokolula umzimba) ukuze ulungele ezinye iimvavanyo.
Siza kukucela ukuba uhambe iimitha ezimbalwa ngeli lixa sirekhoda yonke into ngevidiyo. Siza kuthatha imilinganiselo yonyawo lwakho ngeli lixa umile naxa uhleli phantsi. Sakulandela ngokwenza uvavanyo lokubhalansa. Kuza kufuneka ume ngomlenze omnye imizuzwana engama-30.Emva koko kuza kufuneka uzame ukuhamba ubuya umva kumaplangwe amathathu anemilinganiselo eyahlukileyo, ukwenze oku ka-3.
Uza kucelwa ukuba utsibe uye phambili, utsibele kude kangangoko unakho ka-3 ze uphinde utsibele emacaleni kaninzi kangangoko unakho ngemizuzwana eli-15. Oku uza kwenza kabini.
Okulandelayo uza kuhamba, unkcunkce ze ubaleke iimitha ezingama-20 ngeli lixa urekhodwa ngevidiyo. Sifuna ukubona ukuba ulubeka njani unyawo phantsi ngeli lixa ubaleka. Kukubaleka qha okuya kwenziwa kabini ukanti liya kuthi libhalwe phantsi ixesha olithathileyo ukugqiba oku.
Okokugqibela siya kurekhoda ixesha lakho ngeli lixa ubaleka iimitha ezi-5, ujike uphinde ubaleke amatyeli ali-10.

Ikhona into embi enokundehlela?
Akhonto imbi enokwehlela kolu phando. Uza kubaleka imigama emifutshane ze uxhume kathathu. Into nje enokwehlela kukuba izihlunu zakho zisenokudinwa.
Siza kubonisa yonke into ekufuneka uyenze.

Ukhona umntu oza kwazi ukuba ndikolu phononongo?
Liqela elisebenza kule projekthi kuphela eliza kukwazi ukuba uyinxalenye yolu phando. Iziphumo zakho ngqo ziya kwaziwa nguElbé kuphela.

Ndingathetha nabani malunga nolu phononongo?
Ukuba unemibuzo okanye ufuna ukuthetha nomntu malunga nolu phando, ungaqhagamshelana: noElbé de Villiers (kwiselula 084 515 7642; i-imeyili edup@sun.ac.za) okanye noGqr Ranel Venter (kwiselula 083 309 2894; i-imeyili rev@sun.ac.za).

Kuza kwenzeka ntoni xa ndingafuni ukuyenza le nto?
Akukho mntu unokukunyanzela ukuba ube yinxalenye yolu phando Ukuba akufuni kukwenza oku, akunyanzelekanga ukuba ukwenze. Nokuba abazali bakho bakuvumele baze basayina ifomu, awunyanzelekanga ukuba ukwenze oku.

Ukuba uthe ufuna ukuba yinxalenye yophando, ze kamva ugqibe kwelokuba awusafuni, akukho iza kwenzeka kuwe, ungavele nje uyeke ukuba yinxalenye yalo.

Uyaluqonda olu phononongo lophando yaye uzimisele ukuthatha inxaxheba kulo?

EWE HAYI

Ngaba umphandi uyiphendule yonke imibuzo yakho?

EWE HAYI

Uyaqonda ukuba ungarhoxa kolu phononongo nanini na?

EWE HAYI

Utyikityo lomntwana Umhla

UNIVERSITEIT•STELLENBOSCH•UNIVERSITY
jou kennisvennoot • your knowledge partner

IYUNIVESITHI YASESTELLENBOSCH
IMVUME YOKUTHATHA INXAXHEBA KUPHANDO

Iintshukumo zeenyawo – uphando lokuthelekisa abantwana abathanda ukunxiba izihlangu kunye nabo bathanda ukuhamba ngeenyawo

NdinguElbé de Villiers (umfundi owenza izifundo zobugqirha (PhD) kuBunzululwazi beMidlalo kwiSebe leNzululwazi zeMidlalo) kwiYunivesithi yaseStellenbosch. Ndingathanda ukuba umntwana wakho athathe inxaxheba kuphando endilwenzayo. Iziphumo zolu phando ziza kuba yinxalenye yethisisi yam yezifundo zobugqirha zeNzululwazi yeZemidlalo. Umntwana wakho ukhethwe ukuba abe ngomnye wabo baza kuthatha inxaxheba kolu phando kuba isikolo sakhe sithatha inxaxheba nangenxa yokuba enale minyaka yobudala ndiyifunayo.

1. INJONGO YOPHANDO

Injongo yolu phando kukujonga ifuthe izihlangu ezinalo kwiinyawo ezisakhulayo. Ndiza kujonga nokuba ingaba izihlangu zinefuthe na kwindlela umntwana ahamba ngayo.

2. IINKQUBO

Ukuba uyavuma ukuba umntwana wakho athathe inxaxheba kolu phando, umntwana wakho siya kuthi simvavanyele ezi zinto zilandelayo senze nale milinganiselo ilandelayo:

Umlinganiselo wokuba ungakanani na: Siza kumeta ubude bakhe nobunzima bakhe.

Siza kuzalisa iphepha lemibuzo ngezinto azenzayo ngomzimba wakhe: Oku kwenzelwa ukuba kujongwe ukuba umntwana uwusebenzisa kangakanani na umzimba wakhe (uyadlala na).

Iphepha lemibuzo malunga nokunganxibi izihlangu: Oku kuza kwenzelwa ukuba kujongwe ukuba umntwana wakho uye anganxibi izihlangu kangaphi.

Ukuhamba, ukunkcunkca (ukujoga) nokubaleka iimitha ezingama-20: Okokuqala umntwana wakho uza kuhamba, ze ankcunkce ze abaleke kakhulu imitha ezingama-20. Ngeli lixa enza oku uza kurekhodwa kwividiyokhamera. Uza kubaleka kakhulu kabini. Ividiyo ikholo nje ukujonga ukuba iinyawo uzibeka njani emhlabeni ngeli lixa ebaleka.

Iimvavanyo zokubhalansa: Umntwana wakho uza kucelwa ukuba eme ngomlenze omnye imizuzwana engama-30. Oku kuza kwenziwa kathathu. Emva koko uza kucelwa ukuba ahambe ebuya umva kumaplangwe ama-3, anobubanzi obahlukileyo. Oku kuza kwenziwa kathathu kwiplangwe ngalinye.

*Ukutsiba: Umntwana wakho uza kucelwa ukuba atsibele kude kangangoko anako edibanise iinyawo. Kuya kumetwa umgama. Uza kucelwa ukuba utsibe kathathu.Baza kucelwa ukuba batsibele emacaleni kaninzi kangangoko benakho imizuzwana eli-*15*. Kuza kubalwa ukuba batsibe kangaphi yaye oko baza kukwenza kabini.*

Uvavanyo lweentshukumo zomzimba: Olu vavanyo lwenziwa kwiimitha ezi-5 phakathi kweekhowuni ezimbini. Umntwana wakho uza kubaleka phakathi kweekhowuni, emana ebamba phantsi, amatyeli ali-10. Ixesha eliza kuthathwa ngumntwana wakho ukugqiba iigemu ezili-10 liza kubhalwa phantsi. Uza kuluphinda uvavanyo yaye kuya kusetyenziswa elona xesha alenze kakuhle.

Ukumila konyawo: Umntwana wakho uza kucelwa ukuba eme ngemilenze yomibini ephepheni elincinci, kuya kuthi kumetwe ubude benkwali eligophe yonyawo lwakhe, ubude bonyawo nobubanzi balo kusetyenziswa isixhobo sokumeta, icalliper, ngeli lixa emile naxa ehleli phantsi.

3. **IMINGCIPHEKO NOKUNGAZIVA MNANDI OKUNOKUBA KHONA**

Noxa ezinye zezi mvavanyo zisenokungaziwa ngumntwana wakho, zimvavanyo ezilula. Azisayi kwenza umntwana wakho ukuba adinwe kakhulu okanye azive engonwabanga.

4. IINZUZO EZINOKUBA KHONA KWABO BATHATHA INXAXHEBA KOLU PHANDO

Umntwana wakho akasayi kuzuza nto kolu phando.

Olu phando lunenzuzo yokufumana ulwazi kwizifundo zemidlalo, nangakumbi kwindlela izihlangu ezinefuthe ngayo ezinyaweni zabantwana nendlela abahamba ngayo. Iziphumo zingancedisa nabavelisi bezihlangu ukuba bafumane ulwazi lokudizayina izihlangu eziya kunceda ekukhuleni kweenyawo zabantwana.

5. INTLAWULO YOKUTHATHA INXAXHEBA

Umntwana wakho akazi kuhlawulwa ngokuthatha inxaxheba kolu phando.

6. UBUMFIHLO

Naluphi ulwazi olufunyenwe kolu phando noluya kuveza ukuba ngubani na umntwana wakho, luya kugcinwa luyimfihlo yaye luya kuvezwa kuphela xa kufunyenwe imvume yakho okanye xa lufunwa ngabantu basemthethweni. Izinto ezithethiweyo ziya kugcinwa ziyimfihlo ngokuthi zigcinwe kwikhompyutha enegama eliyimfihlo ekufuneka ulifakile ukuze uyivule. Ngumphandi nomhloli wakhe kuphela abaya kuvumeleka ukuba babone ezi nkcukacha. Ngawo onke amaxesha xa kusetyenziswa olu lwazi akuzi kubandakanywa magama abantu.

Ukuba olu phando luyapapashwa, kuza kuthethwa ngalo gabalala – ngamanye amagama kuza kuhlanganiswa lonke ulwazi olufunyenwe kwiqela.

7. UKUTHATHA INXAXHEBA NOKURHOXA

Ungathatha isigqibo sokuba umntwana wakho athathe inxaxheba kolu phando. Ukuba uyavuma ukuba umtwana wakho athathe inxaxheba, usenokuphinda umrhoxise nanini kuphando yaye oku akusayi kumchaphazela kakubi umntwana wakho nangayiphi na indlela. Umphandi angathatha nesigqibo sokumkhupha kuphando umntwana wakho ukuba imeko itsho.

8. IINKCUKACHA ZABAPHANDI

Ukuba unemibuzo onayo ngophando okanye kukho into engakuhlelanga kamnandi, wamkelekile ukuba ungaqhagamshelana nathi:

UElbé de Villiers (kwiselula 084 515 7642; i-imeyili edup@sun.ac.za) okanye uGqr Ranel Venter (kwiselula 083 309 2894; i-imeyili rev@sun.ac.za)

9. AMALUNGELO ABATHATHI-NXAXHEBA KUPHANDO

Ungarhoxisa imvume yakho nanini na, ungamyekisa umntwana wakho ukuba athathe inxaxheba yaye oku akusayi kuba naziphumo zibi. Umntwana wakho akasayi kuphulukana namalungelo akhe ngokuthatha inxaxheba kolu phando. Ukuba unayo nayiphi na imibuzo emalunga namalungelo omntwana wakho njengomthathi-nxaxheba kolu phando, qhagamshelana noNksz Maléne Fouché kwiCandelo loPhuhliso loPhando leYunivesithi yaseStellenbosch [mfouche@sun.ac.za; 021 808 4622].

**ISIGNITSHA YOMZALI / YOMNTU OJONGE UMNTWANA**

Ndilinikwe ithuba lokuba ndibuze imibuzo yaye ndiye ndafumana iimpendulo ezanelisayo.

Ndiyavuma ukuba ________________________________ angathatha inxaxheba kuphando. Ndiyifumene ikopi yale fomu.

____

Igama lomzali/lomntu ojonge umntwana

___________________________________ _____________

Isignitsha yomzali/yomntu ojonge umntwana **Umhla**

<u>Idilesi yendawo:</u>

Inombolo yesitrato negama: ____

Ummandla/Ilokishi:____

Idolophu/Isixeko: ____

UNIVERSITEIT•STELLENBOSCH•UNIVERSITY
jou kennisvennoot • your knowledge partner

IYUNIVESITHI YASESTELLENBOSCH
IMVUME YOKUTHATHA INXAXHEBA KUPHANDO

Iintshukumo zeenyawo – uphando lokuthelekisa abantwana abathanda ukunxiba izihlangu kunye nabo bathanda ukuhamba ngeenyawo

NdinguElbé de Villiers (umfundi owenza izifundo zobugqirha (PhD) kuBunzululwazi beMidlalo kwiSebe leNzululwazi zeMidlalo) kwiYunivesithi yaseStellenbosch. Ndingathanda ukuba uthathe inxaxheba kuphando endilwenzayo. Iziphumo zolu phando ziza kuba yinxalenye yethisisi yam yezifundo zobugqirha zeNzululwazi yeZemidlalo. Ukhethwe ukuba ube ngomnye wabo baza kuthatha inxaxheba kolu phando kuba isikolo sakho sithatha inxaxheba nangenxa yokuba unale minyaka yobudala ndiyifunayo.

1. INJONGO YOPHANDO

Injongo yolu phando kukujonga ifuthe izihlangu ezinalo kwiinyawo ezisakhulayo. Ndiza kujonga nokuba ingaba izihlangu zinefuthe na kwindlela abantwana abahamba ngayo.

2. IINKQUBO

Ukuba uyavuma ukuba uthathe inxaxheba kolu phando, siya kuthi sikuvavanyele ezi zinto zilandelayo senze nale milinganiselo ilandelayo:

*Umlinganiselo wokuba ungakanani na:*Siza kumeta ubude bakho nobunzima bakho.

Siza kuzalisa iphepha lemibuzo ngezinto ozenzayo ngomzimba wakho: Oku kwenzelwa ukuba kujongwe ukuba uwusebenzisa kangakanani na umzimba wakho (uyadlala na).

Iphepha lemibuzo malunga nokunganxibi izihlangu: Oku kuza kwenzelwa ukuqonda ukuba kukangakanani na apho uye uhambe unganxibanga zihlangu.

Ukuhamba, ukunkcunkca nokubaleka iimitha ezingama-20: kuqala uza kuhamba, unkcunkce ze ubaleke kakhulu iimitha ezingama-20. Ngeli lixa usenza oku, uza kurekhodwa ngevidiyo. Uza kucelwa ukuba ubaleke kakhulu kakhulu kabini. Umsebenzi wevidiyo kukujonga nje ukuba uzibeka njani iinyawo phantsi ngeli lixa ubaleka

Ukubaleka iimitha ezili-20: Ngeli lixa ubalekayo, uza kurekhodwa ngevidiyo. Uza kucelwa ukuba oku ukwenze kathathu.

Uvavanyo lwebhalansi: Uza kucelwa ukuba ume ngomlenze omnye kumgangatho ozinzileyo nongazinzanga (sponji) imizuzwana engama-20, kuqala uvule amehlo ze uphinde uwavale. Oku

kuza kwenziwa kathathu. Emva koko uza kucelwa ukuba ume ngemilenze yomibini, kumgangatho ozinzileyo nongazinzanga (sponji), kuqala uvule amehlo ze uphinde awavale, imizuzwana engama-20, noku kuza kwenziwa kathathu.

Ukutsiba: Uza kucelwa ukuba utsibe kangangoko unakho udibanise iinyawo zombini. Umgama owutsibileyo uza kumetwa. Oku uza kukwenza kathathu. Uza kucelwa kananjalo ukuba utsibele emacaleni kaninzi kangangoko unakho ukwenze oko ngemizuzwana eli-15. Amatyeli otsibe ngawo aza kubalwa yaye oko uza kwenza kabini.

Uvavanyo lweentshukumo zomzimba: Ngeli lixa ukwenza oku kumgama weemitha ezili-15, uza kurekhodwa ngevidiyo. Kuya kuthathwa olo vavanyo lungcono kwiimvavanyo ezimbini.

Ubume bonyawo: Uza kucelwa ukuba ume ngomlenze omnye kwipleyiti, ze kujongwe ubude nobubanzi bonyawo lwakho nendlela unyawo olume ngayo.

3. **IMINGCIPHEKO NOKUNGAZIVA MNANDI OKUNOKUBA KHONA**

Noxa ezinye zezi mvavanyo usenokungazazi, zimvavanyo ezilula. Azisayi kwenza ukuba udinwe kakhulu okanye uzive engonwabanga.

4. IINZUZO EZINOKUBA KHONA KWABO BATHATHA INXAXHEBA KOLU PHANDO

Akusayi kuzuza nto kolu phando.

Olu phando lunenzuzo yokufumana ulwazi kwizifundo zemidlalo, nangakumbi kwindlela izihlangu ezinefuthe ngayo ezinyaweni zabantwana nendlela abahamba ngayo. Iziphumo zingancedisa nabavelisi bezihlangu ukuba bafumane ulwazi lokudizayina izihlangu eziya kunceda ekukhuleni kweenyawo zabantwana.

5. INTLAWULO YOKUTHATHA INXAXHEBA

Awuzi kuhlawulwa ngokuthatha inxaxheba kolu phando.

6. UBUMFIHLO

Naluphi ulwazi olufunyenwe kolu phando oluchaza ukuba ungubani na, luya kugcinwa luyimfihlo yaye luya kuvezwa kuphela xa kufunyenwe imvume yakho okanye xa lufunwa ngabantu basemthethweni. Izinto ezithethiweyo ziya kugcinwa ziyimfihlo ngokuthi zigcinwe kwikhompyutha enegama eliyimfihlo ekufuneka ulifakile ukuze uyivule. Ngumphandi nomhloli wakhe kuphela abaya kuvumeleka ukuba babone ezi nkcukacha. Ngawo onke amaxesha xa kusetyenziswa olu lwazi akuzi kubandakanywa magama abantu.

Ukuba olu phando luyapapashwa, kuza kuthethwa ngalo gabalala – ngamanye amagama kuza kuhlanganiswa lonke ulwazi olufunyenwe kwiqela.

7. UKUTHATHA INXAXHEBA NOKURHOXA

Ungathatha isigqibo sokuba uthathe inxaxheba kolu phando. Ukuba uyavuma ukuba uthathe inxaxheba, usenokuphinda urhoxe nanini kuphando yaye oku akusayi kukuchaphazela kakubi nangayiphi na indlela. Umphandi angathatha nesigqibo sokukukhupha kuphando ukuba imeko itsho.

8. IINKCUKACHA ZABAPHANDI

Ukuba unemibuzo onayo ngophando okanye kukho into engakuhlelanga kamnandi, wamkelekile ukuba ungaqhagamshelana nathi:

UElbé de Villiers (kwiselula 084 515 7642; i-imeyili edup@sun.ac.za) okanye uGqr Ranel Venter (kwiselula 083 309 2894; i-imeyili rev@sun.ac.za)

9. AMALUNGELO ABATHATHI-NXAXHEBA KUPHANDO

Ungarhoxisa imvume yakho nanini na, ungayeka u ukuthatha inxaxheba yaye oku akusayi kuba naziphumo zibi. Akusayi kuphulukana namalungelo akho ngokuthatha inxaxheba kolu phando. Ukuba unayo nayiphi na imibuzo emalunga namalungelo akho njengomthathi-nxaxheba kolu phando, qhagamshelana noNksz Maléne Fouché kwiCandelo loPhuhliso loPhando leYunivesithi yaseStellenbosch [mfouche@sun.ac.za; 021 808 4622].

ISIGNITSHA YOMTHATHI-NXAXHEBA

Ndilinikwe ithuba lokuba ndibuze imibuzo yaye ndiye ndafumana iimpendulo ezanelisayo.

Ndiyavuma ukuba mna ________________________________ ndiza kuthatha inxaxheba kuphando. Ndiyifumene ikopi yale fomu.

____

Igama lomthathi-nxaxheba

___________________________________ _____________

Isignitsha yomthathi-nxaxheba **Umhla**

UNIVERSITEIT STELLENBOSCH

INLIGTINGSTUK EN TOESTEMMINGSVORM VIR DEELNEMERS

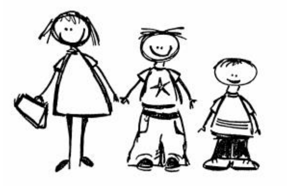

NAAM VAN DIE NAVORSINGSPROJEK: Bewegende voete – ’n studie waar ons skoolkinders wat gewoonlik skoene dra vergelyk met dié wat gewoonlik kaalvoet loop

NAVORSER(S) SE NAAM: Elbé de Villiers

ADRES: Departement Sportwetenskap, Universiteit Stellenbosch

KONTAKNOMMER: 021 808 4735 **/** 084 515 7642

Wat is NAVORSING?
Navorsing is iets wat ons doen om MEER TE LEER oor hoe dinge (en mense) werk. Ons gebruik navorsingsprojekte of -ondersoeke om meer uit te vind oor kinders en tieners en die dinge wat hulle lewe beïnvloed, soos hulle skool, hulle gesin en hulle gesondheid. Ons doen dit omdat ons die wêreld ’n beter plek probeer maak.

Waaroor gaan hierdie navorsingsprojek?
Met hierdie navorsing wil ons kyk of die skoene wat jy dra, die volgende doen:
Die manier waarop jy loop verander
Die vorm van jou voet verander
Jou balans beter maak
Jou verder laat spring

Hoekom vra julle my om aan hierdie navorsingsprojek deel te neem?
Ons wil graag hê dat jy moet deelneem aan die projek, omdat jy in die skool is wat ons gekies het om deel te wees, jy gesond is, jy nie enige beserings het nie, en jy die regte ouderdom is.

Wie doen die navorsing?
My naam is Elbé de Villiers en ek werk by die Universiteit Stellenbosch. Ek is ’n Biokinetikus. Ek gebruik oefening om mense sterker te maak nadat hulle seergekry het of as hulle baie siek was.

Wat sal ek moet doen as ek aan die studie deelneem?
Ons gaan eers kyk hoe lank en hoe swaar jy is.
Daarna gaan ons jou laat opwarm deur liggies te draf en bietjie strekke te doen om jou reg te kry vir die toetse.
Jy gaan 20 meter moet hardloop terwyl jy met 'n videokamera afgeneem word en jou tyd geneem word.
Dan gaan jy 'n op 'n meetapparaat moet staan vir 'n paar sekondes, sodat ons jou voet kan meet.
Ons gaan ook jou balans toets. Jy sal agteruit moet loop op drie verskillende plankies. Dit gaan jy twee keer moet doen.
Volgende gaan ons kyk hoe ver jy met altwee bene gelyktydig kan spring.
Daarna gaan ons kyk hoeveel keer jy sywaarts kan spring in 15 sekondes. Dit moet ook twee keer gedoen word.
Laastens gaan ons ook kyk hoe sterk jou handgreep is.

Is daar enigiets wat kan verkeerd gaan?
Jy gaan kort ente hardloop en driekeer spring en jou spiere kan dalk vreemd voel, maar niks kan jou seermaak of niks kan verkeerd gaan nie.
Ons sal ook vir jou mooi wys hoe om alles te doen.

Sal ander mense weet ek neem aan die projek deel?
Niemand hoef te weet dat jy aan die studie deelneem nie en niemand anders, behalwe Elbé, sal weet hoe jy met die toetse gevaar het nie.

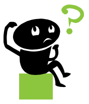

Met wie kan ek oor die projek gesels?
As jy enige vrae het oor die projek of as jy met iemand wil gesels kan jy vir Elbé de Villiers (selfoon: 084 515 7642; e-pos: edup@sun.ac.za) of Dr Ranel Venter (selfoon: 083 309 2894; e-pos: rev@sun.ac.za) kontak.

Wat gebeur as ek nie wil deelneem nie?
Jy hoef net deel te neem aan die projek as jy wil. Jy gaan nie gedwing word nie en dit maak nie saak as jou ouers gesê het jy mag nie, en as jy nie wil nie, hoef jy nie.

As jy wel gesê het jy wil deelneem en jy sien later jy is nie lus nie, kan jy enige tyd vir my sê en dan kan jy ophou deelneem aan die projek.

Verstaan jy waaroor hierdie navorsing gaan, en sal jy aan die projek deelneem?

JA	NEE

Het die navorser ál jou vrae beantwoord?

JA	NEE

Verstaan jy dat jy kan OPHOU deelneem net wanneer jy wil?

JA	NEE

____________________________   _______________________

Kind se handtekening   Datum

UNIVERSITEIT·STELLENBOSCH·UNIVERSITY
jou kennisvennoot • your knowledge partner

UNIVERSITEIT STELLENBOSCH
TOESTEMMING TOT DEELNAME AAN NAVORSING

Bewegende voete – ’n vergelykende studie van skoolkinders wat gewoonlik skoene dra teenoor dié wat gewoonlik kaalvoet loop

Ek is Elbé de Villiers (’n PhD-student in Sportwetenskap) van die Departement Sportwetenskap aan die Universiteit Stellenbosch. Ek nooi u kind om deel te neem aan my navorsingstudie. Die resultate van die studie sal deel uitmaak van die tesis vir my doktorsgraad in Sportwetenskap. U kind is as ’n moontlike studiedeelnemer gekies omdat hy/sy in een van die deelnemerskole is en ook die regte ouderdom is.

1. DOEL VAN DIE STUDIE

Die hoofdoel van hierdie studie is om te bepaal watter effek skoene op die ontwikkelende voet het. Ek sal ook vasstel of skoene kinders se bewegingsvermoë beïnvloed.

2. PROSEDURES

Indien u instem dat u kind aan hierdie studie kan deelneem, sal u kind die volgende toetse en metings ondergaan:

Antropometriese meting: U kind se lengte en gewig sal gemeet word.

Invul van ’n vraelys oor fisiese aktiwiteit: Dít word gedoen om te bepaal hoe aktief u kind is.

Invul van ’n vraelys oor kaalvoetgewoontes: Hiermee wil ons agterkom hoe gereeld u kind kaalvoet is.

Draf en hardloop oor 20 meter: Terwyl u kind draf en hardloop sal hy/sy met ’n videokamera afgeneem word. Die video word geneem om te kyk hoe u kind se voet neergesit word tydens die verskillende situasies. Die tyd wat dit u kind neem om die 20 meter te hardloop sal geneem word en hy/sy sal gevra word om dit twee keer te doen met en sonder skoene.

Balanstoetse: Die kind sal gevra word om agteruit te loop op drie verskillende plankies, elkeen met ’n ander breedte. Dit moet twee keer elk gedoen word met en sonder skoene.

Spronge: U kind sal gevra word om so ver as moontlik met albei voete tegelyk te spring. Die afstand sal gemeet word. U kind sal die sprong drie keer doen, met en sonder skoene.
Na die verspring sal u kind gevra word om so veel keer as moontlik in 15 sekondes sywaarts te spring. Dit sal twee keer herhaal word en die beste een sal gebruik word, met en sonder skoene.

Handgreep: Die krag van albei u kind se hande sal gemeet word met 'n handgreepkaliper.

Voetvorm: U kind sal gevra word om kaalvoet op 'n voetmetingsapparaat te staan waar u kind se voetlengte en -breedte sowel as die hoogte van sy/haar voetbrug bepaal sal word.

3. MOONTLIKE RISIKO'S EN ONGEMAK

Hoewel van die toetse dalk onbekend sal wees vir u kind, is dit eenvoudige toetse. Dit behoort nie u kind buitengewoon moeg te maak of ongemak te veroorsaak nie.

4. MOONTLIKE VOORDELE VIR STUDIEDEELNEMERS EN/OF DIE SAMELEWING

U kind sal geen direkte voordeel uit die studie trek nie.

Die studie hou egter wel voordele in vir kennis op die gebied van sportwetenskap en veral oor die uitwerking van skoene op kinders se voete en bewegingsvermoë. Die resultate kan skoenvervaardigers ook moontlik in die toekoms die nodige kennis gee om skoene te ontwerp wat voordelig is vir die ontwikkeling van kinders se voete.

5. VERGOEDING VIR DEELNAME

U kind sal nie vir deelname aan hierdie studie betaal word nie.

6. VERTROULIKHEID

Enige inligting wat in verband met hierdie studie bekom word en u kind se identiteit verklap, sal vertroulik bly en slegs met u toestemming of ingevolge wetsvereistes bekend gemaak word. Vertroulikheid sal gehandhaaf word deur die data op 'n persoonlike rekenaar met 'n wagwoord te berg. Slegs die navorser en die studieleier sal na die data kan kyk. Die data sal te alle tye anoniem hanteer word.

Indien die navorsing gepubliseer word, sal die data in die algemeen – met ander woorde vir die groep in die geheel – bespreek word.

7. DEELNAME EN ONTTREKKING

U kan kies of u kind aan hierdie studie mag deelneem of nie. Indien u aanbied dat u kind kan deelneem, kan u hom/haar steeds in enige stadium onttrek sonder dat dit enige gevolge vir u kind sal inhou. Die navorser kan ook besluit om u kind aan die studie te onttrek indien omstandighede dit vereis.

8. BESONDERHEDE VAN NAVORSERS

As u enige vrae oor die navorsing het of as enigiets daarvan u pla, kontak ons gerus:

Elbé de Villiers (selfoon 084 515 7642; e-pos edup@sun.ac.za) of dr Ranel Venter (selfoon 083 309 2894; e-pos rev@sun.ac.za)

9. REGTE VAN NAVORSINGSDEELNEMERS

U kan in enige stadium u toestemming terugtrek en u kind se deelname staak, sonder enige nadelige gevolge. U kind doen nie afstand van enige wettige aansprake of regte deur aan hierdie navorsingstudie deel te neem nie. Vir enige vrae oor u kind se regte as studiedeelnemer, skakel met me Maléne Fouché in die Universiteit Stellenbosch se Afdeling Navorsingsontwikkeling [mfouche@sun.ac.za; 021 808 4622].

HANDTEKENING VAN OUER / VOOG

Ek het geleentheid gekry om vrae te vra, en dit is bevredigend beantwoord.

Ek stem in dat ___________________________ aan hierdie studie kan deelneem. Ek het 'n afskrif van hierdie vorm ontvang.

____

Naam van ouer/voog

______________________________ ____________

Handtekening van ouer/voog **Datum**

<u>Woonadres:</u>

Straatnaam en nommer: ______________________________

Voorstad / area: ______________________________

Stad / Dorp: ______________________________

UNIVERSITEIT·STELLENBOSCH·UNIVERSITY
jou kennisvennoot • your knowledge partner

UNIVERSITEIT STELLENBOSCH
TOESTEMMING TOT DEELNAME AAN NAVORSING

Bewegende voete – ’n vergelykende studie van skoolkinders wat gewoonlik skoene dra teenoor dié wat gewoonlik kaalvoet loop

Ek is Elbé de Villiers (’n PhD-student in Sportwetenskap) van die Departement Sportwetenskap aan die Universiteit Stellenbosch. Ek nooi jou om deel te neem aan my navorsingstudie. Die resultate van die studie sal deel uitmaak van die tesis vir my doktorsgraad in Sportwetenskap. Jy is as ’n moontlike studiedeelnemer gekies omdat jy in een van die deelnemerskole is en ook die regte ouderdom is.

1. DOEL VAN DIE STUDIE

Die hoofdoel van hierdie studie is om te bepaal watter effek skoene op die ontwikkelende voet het. Ek sal ook vasstel of skoene kinders se bewegingsvermoë beïnvloed.

2. PROSEDURES

Indien jy instem om aan hierdie studie deel te neem, sal jy die volgende toetse en metings ondergaan:

Antropometriese meting: Jou lengte en gewig sal gemeet word.

Invul van ’n vraelys oor fisiese aktiwiteit: Dít word gedoen om te bepaal hoe aktief jy is.

Invul van ’n vraelys oor kaalvoetgewoontes: Hiermee wil ons agterkom hoe gereeld jy kaalvoet is.

Draf en hardloop oor 20 meter: Terwyl jy draf en hardloop sal jy met ’n videokamera afgeneem word. Die video word geneem om te kyk hoe jy jou voet neersit tydens die verskillende situasies. Die tyd wat dit jou neem om die 20 meter te hardloop sal geneem word en jy sal gevra word om dit twee keer te doen, met en sonder skoene.

Balanstoetse: Jy sal gevra word om agteruit te loop op drie verskillende plankies, elkeen met ’n ander breedte. Dit moet ook twee keer elk gedoen word, met en sonder skoene.

Spronge: Jy sal so ver as moontlik met albei voete tegelyk probeer spring. Die afstand sal gemeet word. Jy sal die sprong drie keer doen, met en sonder skoene.
Na die verspring sal jy gevra word om so veel keer as moontlik in 15 sekondes sywaarts te spring. Dit sal twee keer herhaal word en die beste een van die twee sal gebruik word, met en sonder skoene.

UNIVERSITEIT•STELLENBOSCH•UNIVERSITY
jou kennisvennoot • your knowledge partner

STELLENBOSCH UNIVERSITY
CONSENT TO PARTICIPATE IN RESEARCH

Moving feet – a comparative study of school children who normally wear shoes and those who normally walk barefoot

I am Elbé de Villiers (a PhD student in Sport Science) of the Department of Sport Science at Stellenbosch University. I would like to invite your child to participate in my research study. The results of the study will form part of the thesis for my doctoral degree in Sport Science. Your child has been chosen as a possible participant in the study because he/she is in one of the participant schools and also is of the right age.

1. PURPOSE OF THE STUDY

The main purpose of this study is to determine the effect that shoes have on the developing foot. I will also determine whether shoes influence children's ability to move.

2. PROCEDURES

If you agree that your child may take part in this study, your child will have to undergo the following tests and measurements:

Anthropometric measurement: Your child's length and weight will be measured.

Complete a questionnaire on physical activity: This is done to determine how active your child is.

Jogging and running for 20 metres: While your child runs, he/she will be recorded on a video camera. The child will be asked to do this three times with and without shoes. The video is just to determine how your child lands with his feet while running.

Balance tests: Your child will be asked to walk backwards on three different sized bars. This will be done twice on each bar with and without shoes.

Jumps: Your child will be asked to jump as far as he/she can with both feet together. The distance will be measured. Your child will do this jump three times with and without shoes.
Next your child will be asked to jump sideways as many times as possible in 15 seconds. They will do it twice with and without shoes.

Foot shape: Your child will be asked to walk over a platform with a pressure plate embedded in it. They will also have to stand on a foot measuring platform, which then will determine the child's foot length and breadth as well as the height of his/her foot bridge while standing and seated.

Grip strength: Your child's grip strength will be determined by using a hand grip calliper.

3. POTENTIAL RISKS AND DISCOMFORT

Although some of the tests might be unknown to your child, they are simple tests. They should not make your child exceptionally tired or cause any discomfort.

4. POTENTIAL BENEFITS FOR STUDY PARTICIPANTS AND/OR SOCIETY

Your child will gain no direct benefit from the study.

The study does hold benefits for knowledge in the field of sport science, however, and specifically on the effect of shoes on children's feet and their ability to move. The results could possibly also provide shoe manufacturers with the necessary knowledge in the future to design shoes that are beneficial for the development of children's feet.

5. REMUNERATION FOR PARTICIPATION

Your child will not be paid for participation in this study.

6. CONFIDENTIALITY

Any information that is obtained in connection with this study and that could reveal your child's identity will remain confidential and will only be revealed with your consent or if required by law. Confidentiality will be maintained by storing the data on a personal computer with a password. Only the researcher and the supervisor will be able to look at the data. The data will be dealt with anonymously at all times.

If the research should be published, the data will be discussed in general – in other words for the group as a whole.

7. PARTICIPATION AND WITHDRAWAL

You can decide whether or not your child may participate in this study. If you offer that your child may participate, you may still withdraw him/her from the study at any stage without this holding any negative consequences for your child. The researcher could also decide to remove your child from the study should circumstances require this.

8. DETAILS OF RESEARCHERS

If you have any questions on the research or if anything about it bothers you, you are welcome to contact us:

Elbé de Villiers (cell phone 084 515 7642; e-mail edup@sun.ac.za) or Dr Ranel Venter (cell phone 083 309 2894; e-mail rev@sun.ac.za)

9. RIGHTS OF RESEARCH PARTICIPANTS

You may withdraw your consent at any stage and discontinue your child's participation, without any negative consequences. Your child will not waive any legal claims or rights by taking part in this research study. For any questions about your child's rights as a study participant, contact Ms Maléne Fouché at the Stellenbosch University Division for Research Development [mfouche@sun.ac.za; 021 808 4622].

SIGNATURE OF PARENT / GUARDIAN

I was given a copy of the letter with information.
I was given the opportunity to ask questions, and they were answered satisfactorily.

I consent that ___________________________________ may participate in this study. I have received a copy of this form.

____
Name of parent/guardian

______________________________________ ________________
Signature of parent/guardian **Date**

<u>Physical Address:</u>

Street number and name:____

Area / Suburb: ____

Town / City: ____

UNIVERSITEIT•STELLENBOSCH•UNIVERSITY
jou kennisvennoot • your knowledge partner

STELLENBOSCH UNIVERSITY
CONSENT TO PARTICIPATE IN RESEARCH

Moving feet – a comparative study of school children who normally wear shoes and those who normally walk barefoot

I am Elbé de Villiers (a PhD student in Sport Science) of the Department of Sport Science at Stellenbosch University. I would like to invite you to participate in my research study. The results of the study will form part of the thesis for my doctoral degree in Sport Science. You have been chosen as a possible participant in the study because you are in one of the participant schools and also are of the right age.

1. PURPOSE OF THE STUDY

The main purpose of this study is to determine the effect that shoes have on the developing foot. I will also determine whether shoes influence children's ability to move.

2. PROCEDURES

If you agree to take part in this study, you will have to undergo the following tests and measurements:

Anthropometric measurement: Your length and weight will be measured.

Complete a questionnaire on physical activity: This is done to determine how active you are.

Questionnaire on being barefoot: This will be done to determine how often you are barefoot.

Jogging and running for 20 metres: First you will jog and then sprint for 20 metres. While doing this, you will be recorded on a video camera. You will be asked to do the sprinting twice with and without shoes. The video is just to determine how you land with your feet while running.

Balance tests: You will be asked to walk backwards on three different sized bars. This will be done twice on each bar with and without shoes.

Jumping: You will be asked to jump as far as you can with both feet together. The distance will be measured. You will do this jump three times with and without shoes.
With the next jump, you will have to jump sideways as many times as possible in 15 seconds. The jumps will be counted and you will do it twice with and without shoes.

Foot shape: You will be asked to walk over a platform with a pressure plate embedded in it.
You would also have to stand with both legs on a foot measuring platform and your arch height, foot length and foot width will be measured by a calliper while you are standing and being seated.

Grip strength: Your grip strength will be determined by using a hand grip calliper.

3. POTENTIAL RISKS AND DISCOMFORT

Although some of the tests might be unknown to you, they are simple tests. They should not make you exceptionally tired or cause any discomfort.

4. POTENTIAL BENEFITS FOR STUDY PARTICIPANTS AND/OR SOCIETY

You will gain no direct benefit from the study.

The study does hold benefits for knowledge in the field of sport science, however, and specifically on the effect of shoes on children's feet and their ability to move. The results could possibly also provide shoe manufacturers with the necessary knowledge in the future to design shoes that are beneficial for the development of children's feet.

5. REMUNERATION FOR PARTICIPATION

You will not be paid for participation in this study.

6. CONFIDENTIALITY

Any information that is obtained in connection with this study and that could reveal your identity will remain confidential and will only be revealed with your consent or if required by law. Confidentiality will be maintained by storing the data on a personal computer with a password. Only the researcher and the supervisor will be able to look at the data. The data will be dealt with anonymously at all times.

If the research should be published, the data will be discussed in general – in other words for the group as a whole.

7. PARTICIPATION AND WITHDRAWAL

You can decide whether or not you want to participate in this study. If you offer that you will participate, you may still withdraw from the study at any stage without this holding any negative consequences for you. The researcher could also decide to remove you from the study should circumstances require this.

8. DETAILS OF RESEARCHERS

If you have any questions on the research or if anything about it bothers you, you are welcome to contact us:

Elbé de Villiers (cell phone 084 515 7642; e-mail edup@sun.ac.za) or Dr Ranel Venter (cell phone 083 309 2894; e-mail rev@sun.ac.za)

9. RIGHTS OF RESEARCH PARTICIPANTS

You may withdraw your consent at any stage and discontinue your participation, without any negative consequences. You will not waive any legal claims or rights by taking part in this research study. For any questions about your rights as a study participant, contact Ms Maléne Fouché at the Stellenbosch University Division for Research Development [mfouche@sun.ac.za; 021 808 4622].

SIGNATURE OF PARTICIPANT

I was given a copy of the letter with information.
I was given the opportunity to ask questions, and they were answered satisfactorily.

I consent that I, ________________________________ will participate in this study. I have received a copy of this form.

____
Name of participant

___________________________________ ________________
Signature of participant **Date**

IYUNIVESITHI YASESTELLENBOSCH

INCWADANA YEENKCUKACHA ZOMTHATHI-NXAXHEBA NEFOMU YEMVUME

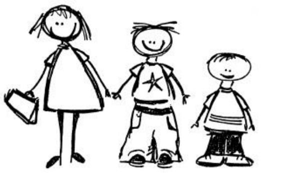

ISIHLOKO SEPROJEKTHI YOPHANDO:
Iintshukumo zeenyawo – uphando lokuthelekisa abantwana abathanda ukunxiba izihlangu kunye nabo bathanda ukuhamba ngeenyawo.

IGAMA LOMPHANDI: Elbé de Villiers

IDILESI: Isebe loBunzulululwazi bezeMidlalo, kwiYunivesithi yaseStellenbosch

INOMBOLO YOQHAGAMSHELWANO: 021,808 (4735)21 938 4735

Luyintoni uPHANDO?
Uphando yinto esiyenzayo ukukhangela **ULWAZI OLUTSHA** malunga nendlela ezisebenza ngayo izinto (nabantu). Senza iiprojekthi zophando ukufumanisa ngakumbi ngabantwana abancinci kunye nabo sebekhulile kunye nezinto ezichaphazela ubomi babo, izikolo zabo, iintsapho zabo nempilo yabo. Sikwenza oku ukuze senze ihlabathi libe yindawo engcono!

Le projekthi yophando imalunga nantoni?
Ngexesha lokwenza le projekthi sifuna ukubona ukuba izihlangu zakho zemihla ngemihla ezi zinaliphi ifuthe ezinyaweni zakho:
Indlela ohamba ngayo
Ubume beenyawo zakho
Indlela obhalansa ngayo
Umgama onokuxhumela kuwo
Ixesha olithathayo ukubaleka umgama omfutshane nokujika uphinde ubuye umva

Bekutheni ukuze ndicelwe ukuba ndithathe inxaxheba kule projekthi yophando?
Ucelwe kuba ungumfundi kwesinye sezikolo esikhethelwe ukuba kwenziwe kuso uphando. Uphilile, awenzakalanga yaye ubudala bakho bobu sifunayo.

Lwenziwa ngubani olu phando?
Igama lam nguElbé de Villiers. Ndingumoluli womzimba osebenza kwiYunivesithi yaseStellenbosch. Umsebenzi wam kukunceda ukuba abantu babe ngcono emva kokufumana umenzakalo, naxa bebesengozini okanye xa bebegula kakhulu. Sibanceda ngokuthi sibenzise imithambo ethile.

Kuza kwenzeka ntoni kum kolu phando?
Kolu phando siza kwenza iimvavanyo ezimbalwa.
Okokuqala siza kumeta ubude bakho nobunzima bakho.
Emva koko siza kwenza imithambo yokuzilungiselela (ukubaleka kancinci nokolula umzimba) ukuze ulungele ezinye iimvavanyo.
Siza kukucela ukuba uhambe iimitha ezimbalwa ngeli lixa sirekhoda yonke into ngevidiyo. Siza kuthatha imilinganiselo yonyawo lwakho ngeli lixa umile naxa uhleli phantsi. Sakulandela ngokwenza uvavanyo lokubhalansa. Kuza kufuneka ume ngomlenze omnye imizuzwana engama-30.Emva koko kuza kufuneka uzame ukuhamba ubuya umva kumaplangwe amathathu anemilinganiselo eyahlukileyo, ukwenze oku ka-3.
Uza kucelwa ukuba utsibe uye phambili, utsibele kude kangangoko unakho ka-3 ze uphinde utsibele emacaleni kaninzi kangangoko unakho ngemizuzwana eli-15. Oku uza kwenza kabini.
Okulandelayo uza kuhamba, unkcunkce ze ubaleke iimitha ezingama-20 ngeli lixa urekhodwa ngevidiyo. Sifuna ukubona ukuba ulubeka njani unyawo phantsi ngeli lixa ubaleka. Kukubaleka qha okuya kwenziwa kabini ukanti liya kuthi libhalwe phantsi ixesha olithathileyo ukugqiba oku.
Okokugqibela siya kurekhoda ixesha lakho ngeli lixa ubaleka iimitha ezi-5, ujike uphinde ubaleke amatyeli ali-10.

Ikhona into embi enokundehlela?
Akhonto imbi enokwehlela kolu phando. Uza kubaleka imigama emifutshane ze uxhume kathathu. Into nje enokwehlela kukuba izihlunu zakho zisenokudinwa.
Siza kubonisa yonke into ekufuneka uyenze.

Ukhona umntu oza kwazi ukuba ndikolu phononongo?
Liqela elisebenza kule projekthi kuphela eliza kukwazi ukuba uyinxalenye yolu phando. Iziphumo zakho ngqo ziya kwaziwa nguElbé kuphela.

Ndingathetha nabani malunga nolu phononongo?
Ukuba unemibuzo okanye ufuna ukuthetha nomntu malunga nolu phando, ungaqhagamshelana: noElbé de Villiers (kwiselula 084 515 7642; i-imeyili edup@sun.ac.za) okanye noGqr Ranel Venter (kwiselula 083 309 2894; i-imeyili rev@sun.ac.za).

Kuza kwenzeka ntoni xa ndingafuni ukuyenza le nto?
Akukho mntu unokukunyanzela ukuba ube yinxalenye yolu phando Ukuba akufuni kukwenza oku, akunyanzelekanga ukuba ukwenze. Nokuba abazali bakho bakuvumele baze basayina ifomu, awunyanzelekanga ukuba ukwenze oku.

Ukuba uthe ufuna ukuba yinxalenye yophando, ze kamva ugqibe kwelokuba awusafuni, akukho iza kwenzeka kuwe, ungavele nje uyeke ukuba yinxalenye yalo.

Uyaluqonda olu phononongo lophando yaye uzimisele ukuthatha inxaxheba kulo?

EWE HAYI

Ngaba umphandi uyiphendule yonke imibuzo yakho?

EWE HAYI

Uyaqonda ukuba ungarhoxa kolu phononongo nanini na?

EWE HAYI

______________________________ ________________________

Utyikityo lomntwana Umhla

UNIVERSITEIT•STELLENBOSCH•UNIVERSITY
jou kennisvennoot • your knowledge partner

IYUNIVESITHI YASESTELLENBOSCH
IMVUME YOKUTHATHA INXAXHEBA KUPHANDO

Iintshukumo zeenyawo – uphando lokuthelekisa abantwana abathanda ukunxiba izihlangu kunye nabo bathanda ukuhamba ngeenyawo

NdinguElbé de Villiers (umfundi owenza izifundo zobugqirha (PhD) kuBunzululwazi beMidlalo kwiSebe leNzululwazi zeMidlalo) kwiYunivesithi yaseStellenbosch. Ndingathanda ukuba umntwana wakho athathe inxaxheba kuphando endilwenzayo. Iziphumo zolu phando ziza kuba yinxalenye yethisisi yam yezifundo zobugqirha zeNzululwazi yeZemidlalo. Umntwana wakho ukhethwe ukuba abe ngomnye wabo baza kuthatha inxaxheba kolu phando kuba isikolo sakhe sithatha inxaxheba nangenxa yokuba enale minyaka yobudala ndiyifunayo.

1. INJONGO YOPHANDO

Injongo yolu phando kukujonga ifuthe izihlangu ezinalo kwiinyawo ezisakhulayo. Ndiza kujonga nokuba ingaba izihlangu zinefuthe na kwindlela umntwana ahamba ngayo.

2. IINKQUBO

Ukuba uyavuma ukuba umntwana wakho athathe inxaxheba kolu phando, umntwana wakho siya kuthi simvavanyele ezi zinto zilandelayo senze nale milinganiselo ilandelayo:

Umlinganiselo wokuba ungakanani na: Siza kumeta ubude bakhe nobunzima bakhe.

Siza kuzalisa iphepha lemibuzo ngezinto azenzayo ngomzimba wakhe: Oku kwenzelwa ukuba kujongwe ukuba umntwana uwusebenzisa kangakanani na umzimba wakhe (uyadlala na).

Iphepha lemibuzo malunga nokunganxibi izihlangu: Oku kuza kwenzelwa ukuba kujongwe ukuba umntwana wakho uye anganxibi izihlangu kangaphi.

Ukuhamba, ukunkcunkca (ukujoga) nokubaleka iimitha ezingama-20: Okokuqala umntwana wakho uza kuhamba, ze ankcunkce ze abaleke kakhulu imitha ezingama-20. Ngeli lixa enza oku uza kurekhodwa kwividiyokhamera. Uza kubaleka kakhulu kabini. Ividiyo ikholo nje ukujonga ukuba iinyawo uzibeka njani emhlabeni ngeli lixa ebaleka.

Iimvavanyo zokubhalansa: Umntwana wakho uza kucelwa ukuba eme ngomlenze omnye imizuzwana engama-30. Oku kuza kwenziwa kathathu. Emva koko uza kucelwa ukuba ahambe ebuya umva kumaplangwe ama-3, anobubanzi obahlukileyo. Oku kuza kwenziwa kathathu kwiplangwe ngalinye.

*Ukutsiba: Umntwana wakho uza kucelwa ukuba atsibele kude kangangoko anako edibanise iinyawo. Kuya kumetwa umgama. Uza kucelwa ukuba utsibe kathathu.Baza kucelwa ukuba batsibele emacaleni kaninzi kangangoko benakho imizuzwana eli-*15*. Kuza kubalwa ukuba batsibe kangaphi yaye oko baza kukwenza kabini.*

Uvavanyo lweentshukumo zomzimba: Olu vavanyo lwenziwa kwiimitha ezi-5 phakathi kweekhowuni ezimbini. Umntwana wakho uza kubaleka phakathi kweekhowuni, emana ebamba phantsi, amatyeli ali-10. Ixesha eliza kuthathwa ngumntwana wakho ukugqiba iigemu ezili-10 liza kubhalwa phantsi. Uza kuluphinda uvavanyo yaye kuya kusetyenziswa elona xesha alenze kakuhle.

Ukumila konyawo: Umntwana wakho uza kucelwa ukuba eme ngemilenze yomibini ephepheni elincinci, kuya kuthi kumetwe ubude benkwali eligophe yonyawo lwakhe, ubude bonyawo nobubanzi balo kusetyenziswa isixhobo sokumeta, icalliper, ngeli lixa emile naxa ehleli phantsi.

3. **IMINGCIPHEKO NOKUNGAZIVA MNANDI OKUNOKUBA KHONA**

Noxa ezinye zezi mvavanyo zisenokungaziwa ngumntwana wakho, zimvavanyo ezilula. Azisayi kwenza umntwana wakho ukuba adinwe kakhulu okanye azive engonwabanga.

4. IINZUZO EZINOKUBA KHONA KWABO BATHATHA INXAXHEBA KOLU PHANDO

Umntwana wakho akasayi kuzuza nto kolu phando.

Olu phando lunenzuzo yokufumana ulwazi kwizifundo zemidlalo, nangakumbi kwindlela izihlangu ezinefuthe ngayo ezinyaweni zabantwana nendlela abahamba ngayo. Iziphumo zingancedisa nabavelisi bezihlangu ukuba bafumane ulwazi lokudizayina izihlangu eziya kunceda ekukhuleni kweenyawo zabantwana.

5. INTLAWULO YOKUTHATHA INXAXHEBA

Umntwana wakho akazi kuhlawulwa ngokuthatha inxaxheba kolu phando.

6. UBUMFIHLO

Naluphi ulwazi olufunyenwe kolu phando noluya kuveza ukuba ngubani na umntwana wakho, luya kugcinwa luyimfihlo yaye luya kuvezwa kuphela xa kufunyenwe imvume yakho okanye xa lufunwa ngabantu basemthethweni. Izinto ezithethiweyo ziya kugcinwa ziyimfihlo ngokuthi zigcinwe kwikhompyutha enegama eliyimfihlo ekufuneka ulifakile ukuze uyivule. Ngumphandi nomhloli wakhe kuphela abaya kuvumeleka ukuba babone ezi nkcukacha. Ngawo onke amaxesha xa kusetyenziswa olu lwazi akuzi kubandakanywa magama abantu.

Ukuba olu phando luyapapashwa, kuza kuthethwa ngalo gabalala – ngamanye amagama kuza kuhlanganiswa lonke ulwazi olufunyenwe kwiqela.

7. UKUTHATHA INXAXHEBA NOKURHOXA

Ungathatha isigqibo sokuba umntwana wakho athathe inxaxheba kolu phando. Ukuba uyavuma ukuba umtwana wakho athathe inxaxheba, usenokuphinda umrhoxise nanini kuphando yaye oku akusayi kumchaphazela kakubi umntwana wakho nangayiphi na indlela. Umphandi angathatha nesigqibo sokumkhupha kuphando umntwana wakho ukuba imeko itsho.

8. IINKCUKACHA ZABAPHANDI

Ukuba unemibuzo onayo ngophando okanye kukho into engakuhlelanga kamnandi, wamkelekile ukuba ungaqhagamshelana nathi:

UElbé de Villiers (kwiselula 084 515 7642; i-imeyili edup@sun.ac.za) okanye uGqr Ranel Venter (kwiselula 083 309 2894; i-imeyili rev@sun.ac.za)

9. AMALUNGELO ABATHATHI-NXAXHEBA KUPHANDO

Ungarhoxisa imvume yakho nanini na, ungamyekisa umntwana wakho ukuba athathe inxaxheba yaye oku akusayi kuba naziphumo zibi. Umntwana wakho akasayi kuphulukana namalungelo akhe ngokuthatha inxaxheba kolu phando. Ukuba unayo nayiphi na imibuzo emalunga namalungelo omntwana wakho njengomthathi-nxaxheba kolu phando, qhagamshelana noNksz Maléne Fouché kwiCandelo loPhuhliso loPhando leYunivesithi yaseStellenbosch [mfouche@sun.ac.za; 021 808 4622].

ISIGNITSHA YOMZALI / YOMNTU OJONGE UMNTWANA

Ndilinikwe ithuba lokuba ndibuze imibuzo yaye ndiye ndafumana iimpendulo ezanelisayo.

Ndiyavuma ukuba ________________________________ angathatha inxaxheba kuphando. Ndiyifumene ikopi yale fomu.

____

Igama lomzali/lomntu ojonge umntwana

___________________________________ _____________

Isignitsha yomzali/yomntu ojonge umntwana **Umhla**

<u>Idilesi yendawo:</u>

Inombolo yesitrato negama: ____

Ummandla/Ilokishi:____

Idolophu/Isixeko: ____

UNIVERSITEIT·STELLENBOSCH·UNIVERSITY
jou kennisvennoot • your knowledge partner

IYUNIVESITHI YASESTELLENBOSCH
IMVUME YOKUTHATHA INXAXHEBA KUPHANDO

Iintshukumo zeenyawo – uphando lokuthelekisa abantwana abathanda ukunxiba izihlangu kunye nabo bathanda ukuhamba ngeenyawo

NdinguElbé de Villiers (umfundi owenza izifundo zobugqirha (PhD) kuBunzululwazi beMidlalo kwiSebe leNzululwazi zeMidlalo) kwiYunivesithi yaseStellenbosch. Ndingathanda ukuba uthathe inxaxheba kuphando endilwenzayo. Iziphumo zolu phando ziza kuba yinxalenye yethisisi yam yezifundo zobugqirha zeNzululwazi yeZemidlalo. Ukhethwe ukuba ube ngomnye wabo baza kuthatha inxaxheba kolu phando kuba isikolo sakho sithatha inxaxheba nangenxa yokuba unale minyaka yobudala ndiyifunayo.

1. INJONGO YOPHANDO

Injongo yolu phando kukujonga ifuthe izihlangu ezinalo kwiinyawo ezisakhulayo. Ndiza kujonga nokuba ingaba izihlangu zinefuthe na kwindlela abantwana abahamba ngayo.

2. IINKQUBO

Ukuba uyavuma ukuba uthathe inxaxheba kolu phando, siya kuthi sikuvavanyele ezi zinto zilandelayo senze nale milinganiselo ilandelayo:

*Umlinganiselo wokuba ungakanani na:*Siza kumeta ubude bakho nobunzima bakho.

Siza kuzalisa iphepha lemibuzo ngezinto ozenzayo ngomzimba wakho: Oku kwenzelwa ukuba kujongwe ukuba uwusebenzisa kangakanani na umzimba wakho (uyadlala na).

Iphepha lemibuzo malunga nokunganxibi izihlangu: Oku kuza kwenzelwa ukuqonda ukuba kukangakanani na apho uye uhambe unganxibanga zihlangu.

Ukuhamba, ukunkcunkca nokubaleka iimitha ezingama-20: kuqala uza kuhamba, unkcunkce ze ubaleke kakhulu iimitha ezingama-20. Ngeli lixa usenza oku, uza kurekhodwa ngevidiyo. Uza kucelwa ukuba ubaleke kakhulu kakhulu kabini. Umsebenzi wevidiyo kukujonga nje ukuba uzibeka njani iinyawo phantsi ngeli lixa ubaleka

Ukubaleka iimitha ezili-20: Ngeli lixa ubalekayo, uza kurekhodwa ngevidiyo. Uza kucelwa ukuba oku ukwenze kathathu.

Uvavanyo lwebhalansi: Uza kucelwa ukuba ume ngomlenze omnye kumgangatho ozinzileyo nongazinzanga (sponji) imizuzwana engama-20, kuqala uvule amehlo ze uphinde uwavale. Oku

kuza kwenziwa kathathu. Emva koko uza kucelwa ukuba ume ngemilenze yomibini, kumgangatho ozinzileyo nongazinzanga (sponji), kuqala uvule amehlo ze uphinde awavale, imizuzwana engama-20, noku kuza kwenziwa kathathu.

Ukutsiba: Uza kucelwa ukuba utsibe kangangoko unakho udibanise iinyawo zombini. Umgama owutsibileyo uza kumetwa. Oku uza kukwenza kathathu. Uza kucelwa kananjalo ukuba utsibele emacaleni kaninzi kangangoko unakho ukwenze oko ngemizuzwana eli-15. Amatyeli otsibe ngawo aza kubalwa yaye oko uza kwenza kabini.

Uvavanyo lweentshukumo zomzimba: Ngeli lixa ukwenza oku kumgama weemitha ezili-15, uza kurekhodwa ngevidiyo. Kuya kuthathwa olo vavanyo lungcono kwiimvavanyo ezimbini.

Ubume bonyawo: Uza kucelwa ukuba ume ngomlenze omnye kwipleyiti, ze kujongwe ubude nobubanzi bonyawo lwakho nendlela unyawo olume ngayo.

3. IMINGCIPHEKO NOKUNGAZIVA MNANDI OKUNOKUBA KHONA

Noxa ezinye zezi mvavanyo usenokungazazi, zimvavanyo ezilula. Azisayi kwenza ukuba udinwe kakhulu okanye uzive engonwabanga.

4. IINZUZO EZINOKUBA KHONA KWABO BATHATHA INXAXHEBA KOLU PHANDO

Akusayi kuzuza nto kolu phando.

Olu phando lunenzuzo yokufumana ulwazi kwizifundo zemidlalo, nangakumbi kwindlela izihlangu ezinefuthe ngayo ezinyaweni zabantwana nendlela abahamba ngayo. Iziphumo zingancedisa nabavelisi bezihlangu ukuba bafumane ulwazi lokudizayina izihlangu eziya kunceda ekukhuleni kweenyawo zabantwana.

5. INTLAWULO YOKUTHATHA INXAXHEBA

Awuzi kuhlawulwa ngokuthatha inxaxheba kolu phando.

6. UBUMFIHLO

Naluphi ulwazi olufunyenwe kolu phando oluchaza ukuba ungubani na, luya kugcinwa luyimfihlo yaye luya kuvezwa kuphela xa kufunyenwe imvume yakho okanye xa lufunwa ngabantu basemthethweni. Izinto ezithethiweyo ziya kugcinwa ziyimfihlo ngokuthi zigcinwe kwikhompyutha enegama eliyimfihlo ekufuneka ulifakile ukuze uyivule. Ngumphandi nomhloli wakhe kuphela abaya kuvumeleka ukuba babone ezi nkcukacha. Ngawo onke amaxesha xa kusetyenziswa olu lwazi akuzi kubandakanywa magama abantu.

Ukuba olu phando luyapapashwa, kuza kuthethwa ngalo gabalala – ngamanye amagama kuza kuhlanganiswa lonke ulwazi olufunyenwe kwiqela.

7. UKUTHATHA INXAXHEBA NOKURHOXA

Ungathatha isigqibo sokuba uthathe inxaxheba kolu phando. Ukuba uyavuma ukuba uthathe inxaxheba, usenokuphinda urhoxe nanini kuphando yaye oku akusayi kukuchaphazela kakubi nangayiphi na indlela. Umphandi angathatha nesigqibo sokukukhupha kuphando ukuba imeko itsho.

8. IINKCUKACHA ZABAPHANDI

Ukuba unemibuzo onayo ngophando okanye kukho into engakuhlelanga kamnandi, wamkelekile ukuba ungaqhagamshelana nathi:

UElbé de Villiers (kwiselula 084 515 7642; i-imeyili edup@sun.ac.za) okanye uGqr Ranel Venter (kwiselula 083 309 2894; i-imeyili rev@sun.ac.za)

9. AMALUNGELO ABATHATHI-NXAXHEBA KUPHANDO

Ungarhoxisa imvume yakho nanini na, ungayeka u ukuthatha inxaxheba yaye oku akusayi kuba naziphumo zibi. Akusayi kuphulukana namalungelo akho ngokuthatha inxaxheba kolu phando. Ukuba unayo nayiphi na imibuzo emalunga namalungelo akho njengomthathi-nxaxheba kolu phando, qhagamshelana noNksz Maléne Fouché kwiCandelo loPhuhliso loPhando leYunivesithi yaseStellenbosch [mfouche@sun.ac.za; 021 808 4622].

**ISIGNITSHA YOMTHATHI-NXAXHEBA**

Ndilinikwe ithuba lokuba ndibuze imibuzo yaye ndiye ndafumana iimpendulo ezanelisayo.

Ndiyavuma ukuba mna ________________________________ ndiza kuthatha inxaxheba kuphando. Ndiyifumene ikopi yale fomu.

____

Igama lomthathi-nxaxheba

__________________________________ ____________

Isignitsha yomthathi-nxaxheba **Umhla**

	UNIVERSITEIT STELLENBOSCH

INLIGTINGSTUK EN TOESTEMMINGSVORM VIR DEELNEMERS

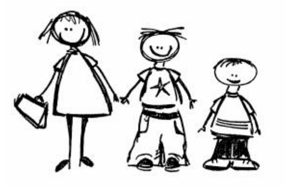

NAAM VAN DIE NAVORSINGSPROJEK: Bewegende voete – 'n studie waar ons skoolkinders wat gewoonlik skoene dra vergelyk met dié wat gewoonlik kaalvoet loop

NAVORSER(S) SE NAAM: Elbé de Villiers

ADRES: Departement Sportwetenskap, Universiteit Stellenbosch

KONTAKNOMMER: 021 808 4735 **/** 084 515 7642

Wat is NAVORSING?
Navorsing is iets wat ons doen om MEER TE LEER oor hoe dinge (en mense) werk. Ons gebruik navorsingsprojekte of -ondersoeke om meer uit te vind oor kinders en tieners en die dinge wat hulle lewe beïnvloed, soos hulle skool, hulle gesin en hulle gesondheid. Ons doen dit omdat ons die wêreld 'n beter plek probeer maak.

Waaroor gaan hierdie navorsingsprojek?
Met hierdie navorsing wil ons kyk of die skoene wat jy dra, die volgende doen:
Die manier waarop jy loop verander
Die vorm van jou voet verander
Jou balans beter maak
Jou verder laat spring

Hoekom vra julle my om aan hierdie navorsingsprojek deel te neem?
Ons wil graag hê dat jy moet deelneem aan die projek, omdat jy in die skool is wat ons gekies het om deel te wees, jy gesond is, jy nie enige beserings het nie, en jy die regte ouderdom is.

Wie doen die navorsing?
My naam is Elbé de Villiers en ek werk by die Universiteit Stellenbosch. Ek is 'n Biokinetikus. Ek gebruik oefening om mense sterker te maak nadat hulle seergekry het of as hulle baie siek was.

Wat sal ek moet doen as ek aan die studie deelneem?
Ons gaan eers kyk hoe lank en hoe swaar jy is.
Daarna gaan ons jou laat opwarm deur liggies te draf en bietjie strekke te doen om jou reg te kry vir die toetse.
Jy gaan 20 meter moet hardloop terwyl jy met 'n videokamera afgeneem word en jou tyd geneem word.
Dan gaan jy 'n op 'n meetapparaat moet staan vir 'n paar sekondes, sodat ons jou voet kan meet.
Ons gaan ook jou balans toets. Jy sal agteruit moet loop op drie verskillende plankies. Dit gaan jy twee keer moet doen.
Volgende gaan ons kyk hoe ver jy met altwee bene gelyktydig kan spring.
Daarna gaan ons kyk hoeveel keer jy sywaarts kan spring in 15 sekondes. Dit moet ook twee keer gedoen word.
Laastens gaan ons ook kyk hoe sterk jou handgreep is.

Is daar enigiets wat kan verkeerd gaan?
Jy gaan kort ente hardloop en driekeer spring en jou spiere kan dalk vreemd voel, maar niks kan jou seermaak of niks kan verkeerd gaan nie.
Ons sal ook vir jou mooi wys hoe om alles te doen.

Sal ander mense weet ek neem aan die projek deel?
Niemand hoef te weet dat jy aan die studie deelneem nie en niemand anders, behalwe Elbé, sal weet hoe jy met die toetse gevaar het nie.

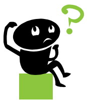

Met wie kan ek oor die projek gesels?
As jy enige vrae het oor die projek of as jy met iemand wil gesels kan jy vir Elbé de Villiers (selfoon: 084 515 7642; e-pos: edup@sun.ac.za) of Dr Ranel Venter (selfoon: 083 309 2894; e-pos: rev@sun.ac.za) kontak.

Wat gebeur as ek nie wil deelneem nie?
Jy hoef net deel te neem aan die projek as jy wil. Jy gaan nie gedwing word nie en dit maak nie saak as jou ouers gesê het jy mag nie, en as jy nie wil nie, hoef jy nie.

As jy wel gesê het jy wil deelneem en jy sien later jy is nie lus nie, kan jy enige tyd vir my sê en dan kan jy ophou deelneem aan die projek.

Verstaan jy waaroor hierdie navorsing gaan, en sal jy aan die projek deelneem?

JA   NEE

Het die navorser ál jou vrae beantwoord?

JA   NEE

Verstaan jy dat jy kan OPHOU deelneem net wanneer jy wil?

JA   NEE

______________________________   ________________________

Kind se handtekening   Datum

UNIVERSITEIT·STELLENBOSCH·UNIVERSITY
jou kennisvennoot • your knowledge partner

UNIVERSITEIT STELLENBOSCH
TOESTEMMING TOT DEELNAME AAN NAVORSING

Bewegende voete – ’n vergelykende studie van skoolkinders wat gewoonlik skoene dra teenoor dié wat gewoonlik kaalvoet loop

Ek is Elbé de Villiers (’n PhD-student in Sportwetenskap) van die Departement Sportwetenskap aan die Universiteit Stellenbosch. Ek nooi u kind om deel te neem aan my navorsingstudie. Die resultate van die studie sal deel uitmaak van die tesis vir my doktorsgraad in Sportwetenskap. U kind is as ’n moontlike studiedeelnemer gekies omdat hy/sy in een van die deelnemerskole is en ook die regte ouderdom is.

1. DOEL VAN DIE STUDIE

Die hoofdoel van hierdie studie is om te bepaal watter effek skoene op die ontwikkelende voet het. Ek sal ook vasstel of skoene kinders se bewegingsvermoë beïnvloed.

2. PROSEDURES

Indien u instem dat u kind aan hierdie studie kan deelneem, sal u kind die volgende toetse en metings ondergaan:

Antropometriese meting: U kind se lengte en gewig sal gemeet word.

Invul van ’n vraelys oor fisiese aktiwiteit: Dít word gedoen om te bepaal hoe aktief u kind is.

Invul van ’n vraelys oor kaalvoetgewoontes: Hiermee wil ons agterkom hoe gereeld u kind kaalvoet is.

Draf en hardloop oor 20 meter: Terwyl u kind draf en hardloop sal hy/sy met ’n videokamera afgeneem word. Die video word geneem om te kyk hoe u kind se voet neergesit word tydens die verskillende situasies. Die tyd wat dit u kind neem om die 20 meter te hardloop sal geneem word en hy/sy sal gevra word om dit twee keer te doen met en sonder skoene.

Balanstoetse: Die kind sal gevra word om agteruit te loop op drie verskillende plankies, elkeen met ’n ander breedte. Dit moet twee keer elk gedoen word met en sonder skoene.

Spronge: U kind sal gevra word om so ver as moontlik met albei voete tegelyk te spring. Die afstand sal gemeet word. U kind sal die sprong drie keer doen, met en sonder skoene.
Na die verspring sal u kind gevra word om so veel keer as moontlik in 15 sekondes sywaarts te spring. Dit sal twee keer herhaal word en die beste een sal gebruik word, met en sonder skoene.

Handgreep: Die krag van albei u kind se hande sal gemeet word met 'n handgreepkaliper.

Voetvorm: U kind sal gevra word om kaalvoet op 'n voetmetingsapparaat te staan waar u kind se voetlengte en -breedte sowel as die hoogte van sy/haar voetbrug bepaal sal word.

3. MOONTLIKE RISIKO'S EN ONGEMAK

Hoewel van die toetse dalk onbekend sal wees vir u kind, is dit eenvoudige toetse. Dit behoort nie u kind buitengewoon moeg te maak of ongemak te veroorsaak nie.

4. MOONTLIKE VOORDELE VIR STUDIEDEELNEMERS EN/OF DIE SAMELEWING

U kind sal geen direkte voordeel uit die studie trek nie.

Die studie hou egter wel voordele in vir kennis op die gebied van sportwetenskap en veral oor die uitwerking van skoene op kinders se voete en bewegingsvermoë. Die resultate kan skoenvervaardigers ook moontlik in die toekoms die nodige kennis gee om skoene te ontwerp wat voordelig is vir die ontwikkeling van kinders se voete.

5. VERGOEDING VIR DEELNAME

U kind sal nie vir deelname aan hierdie studie betaal word nie.

6. VERTROULIKHEID

Enige inligting wat in verband met hierdie studie bekom word en u kind se identiteit verklap, sal vertroulik bly en slegs met u toestemming of ingevolge wetsvereistes bekend gemaak word. Vertroulikheid sal gehandhaaf word deur die data op 'n persoonlike rekenaar met 'n wagwoord te berg. Slegs die navorser en die studieleier sal na die data kan kyk. Die data sal te alle tye anoniem hanteer word.

Indien die navorsing gepubliseer word, sal die data in die algemeen – met ander woorde vir die groep in die geheel – bespreek word.

7. DEELNAME EN ONTTREKKING

U kan kies of u kind aan hierdie studie mag deelneem of nie. Indien u aanbied dat u kind kan deelneem, kan u hom/haar steeds in enige stadium onttrek sonder dat dit enige gevolge vir u kind sal inhou. Die navorser kan ook besluit om u kind aan die studie te onttrek indien omstandighede dit vereis.

8. BESONDERHEDE VAN NAVORSERS

As u enige vrae oor die navorsing het of as enigiets daarvan u pla, kontak ons gerus:

Elbé de Villiers (selfoon 084 515 7642; e-pos edup@sun.ac.za) of dr Ranel Venter (selfoon 083 309 2894; e-pos rev@sun.ac.za)

9. REGTE VAN NAVORSINGSDEELNEMERS

U kan in enige stadium u toestemming terugtrek en u kind se deelname staak, sonder enige nadelige gevolge. U kind doen nie afstand van enige wettige aansprake of regte deur aan hierdie navorsingstudie deel te neem nie. Vir enige vrae oor u kind se regte as studiedeelnemer, skakel met me Maléne Fouché in die Universiteit Stellenbosch se Afdeling Navorsingsontwikkeling [mfouche@sun.ac.za; 021 808 4622].

HANDTEKENING VAN OUER / VOOG

Ek het geleentheid gekry om vrae te vra, en dit is bevredigend beantwoord.

Ek stem in dat ______________________________ aan hierdie studie kan deelneem. Ek het 'n afskrif van hierdie vorm ontvang.

____
Naam van ouer/voog

__________________________________ ______________
Handtekening van ouer/voog **Datum**

Woonadres:

Straatnaam en nommer: ____

Voorstad / area: ____

Stad / Dorp: ____

UNIVERSITEIT·STELLENBOSCH·UNIVERSITY
jou kennisvennoot • your knowledge partner

UNIVERSITEIT STELLENBOSCH
TOESTEMMING TOT DEELNAME AAN NAVORSING

Bewegende voete – 'n vergelykende studie van skoolkinders wat gewoonlik skoene dra teenoor dié wat gewoonlik kaalvoet loop

Ek is Elbé de Villiers ('n PhD-student in Sportwetenskap) van die Departement Sportwetenskap aan die Universiteit Stellenbosch. Ek nooi jou om deel te neem aan my navorsingstudie. Die resultate van die studie sal deel uitmaak van die tesis vir my doktorsgraad in Sportwetenskap. Jy is as 'n moontlike studiedeelnemer gekies omdat jy in een van die deelnemerskole is en ook die regte ouderdom is.

1. DOEL VAN DIE STUDIE

Die hoofdoel van hierdie studie is om te bepaal watter effek skoene op die ontwikkelende voet het. Ek sal ook vasstel of skoene kinders se bewegingsvermoë beïnvloed.

2. PROSEDURES

Indien jy instem om aan hierdie studie deel te neem, sal jy die volgende toetse en metings ondergaan:

Antropometriese meting: Jou lengte en gewig sal gemeet word.

Invul van 'n vraelys oor fisiese aktiwiteit: Dít word gedoen om te bepaal hoe aktief jy is.

Invul van 'n vraelys oor kaalvoetgewoontes: Hiermee wil ons agterkom hoe gereeld jy kaalvoet is.

Draf en hardloop oor 20 meter: Terwyl jy draf en hardloop sal jy met 'n videokamera afgeneem word. Die video word geneem om te kyk hoe jy jou voet neersit tydens die verskillende situasies. Die tyd wat dit jou neem om die 20 meter te hardloop sal geneem word en jy sal gevra word om dit twee keer te doen, met en sonder skoene.

Balanstoetse: Jy sal gevra word om agteruit te loop op drie verskillende plankies, elkeen met 'n ander breedte. Dit moet ook twee keer elk gedoen word, met en sonder skoene.

Spronge: Jy sal so ver as moontlik met albei voete tegelyk probeer spring. Die afstand sal gemeet word. Jy sal die sprong drie keer doen, met en sonder skoene.
Na die verspring sal jy gevra word om so veel keer as moontlik in 15 sekondes sywaarts te spring. Dit sal twee keer herhaal word en die beste een van die twee sal gebruik word, met en sonder skoene.
