# Supplementary material for: The effects of being habitually barefoot on foot mechanics and motor performance in children and adolescents aged 6–18 years: study protocol for a multicenter cross-sectional study (Barefoot LIFE project)
Source: J Foot Ankle Res. 2016 Sep 2;9(1):36. doi: 10.1186/s13047-016-0166-1 (PMC5010736; doi:10.1186/s13047-016-0166-1)
Supplement: Additional file 2: — Barefoot questionnaire for primary and secondary school. (PDF 67 kb) [file 13047_2016_166_MOESM2_ESM.pdf]

Name:

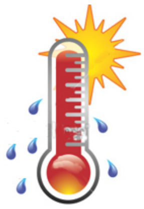

Questionnaire on how often you wear shoes

Circle how often you wear shoes at school:

Almost never	*Half of the time*	*Almost always*

Circle how often you wear shoes during sport activities:

Almost never	*Often*	*Almost always*

Circle how often you wear shoes in and around the home:

Almost never	*Often*	*Almost always*

SECONDARY SCHOOL

Circle how often you wore shoes at primary school:

Almost never	*Often*	*Almost always*

Circle how often you wore shoes during sport activities at primary school:

Almost never	*Often*	*Almost always*

Circle how often you wore shoes in and around the home while you were at primary school:

Almost never	*Often*	*Almost always*

Baie dankie! Thank you! Enkosi!
