# Supplementary figures and images for: The effects of being habitually barefoot on foot mechanics and motor performance in children and adolescents aged 6–18 years: study protocol for a multicenter cross-sectional study (Barefoot LIFE project)
Source: J Foot Ankle Res. 2016 Sep 2;9(1):36. doi: 10.1186/s13047-016-0166-1 (PMC5010736; doi:10.1186/s13047-016-0166-1)

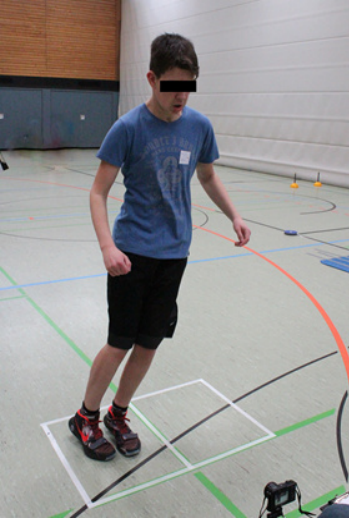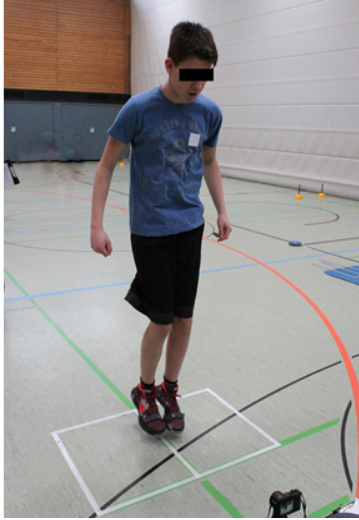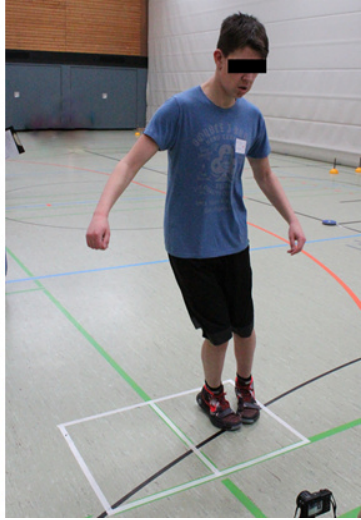

Supplement: Additional file 3: — Participant at the lateral jumping station. (PDF 175 kb) [file 13047_2016_166_MOESM3_ESM.pdf]

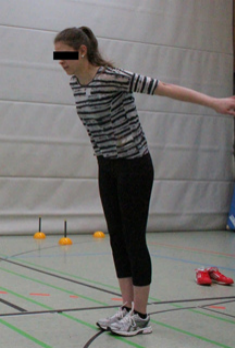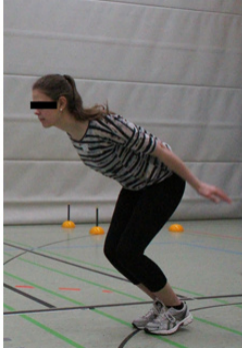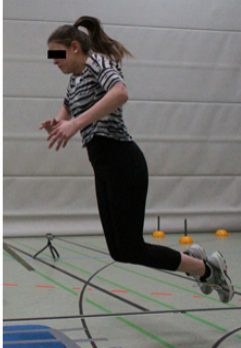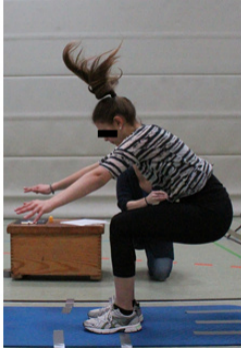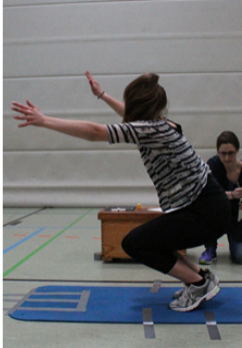

Supplement: Additional file 4: — Participant at the standing long jump station. (PDF 175 kb) [file 13047_2016_166_MOESM4_ESM.pdf]

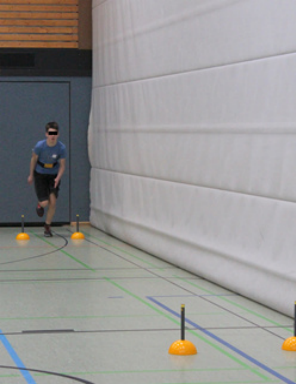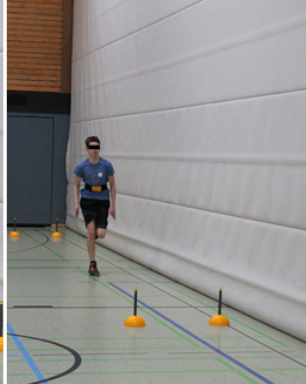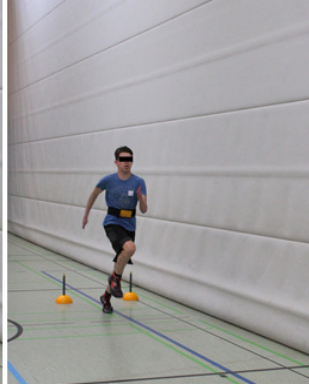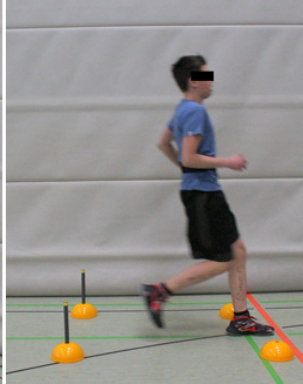

Supplement: Additional file 5: — Participant at the 20 m sprinting station. (PDF 134 kb) [file 13047_2016_166_MOESM5_ESM.pdf]

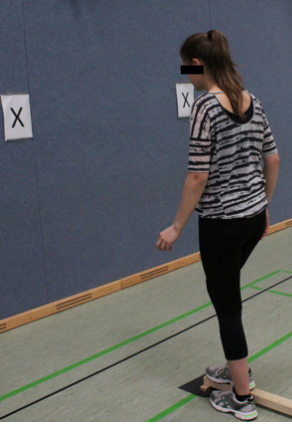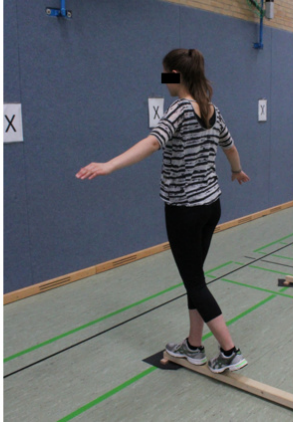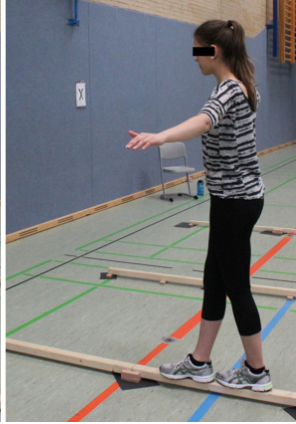

Supplement: Additional file 6: — Participant at the backwards balancing station. (PDF 183 kb) [file 13047_2016_166_MOESM6_ESM.pdf]
